# Cytotoxicity study of the interleukin-12-expressing recombinant Newcastle disease virus strain, rAF-IL12, towards CT26 colon cancer cells in vitro and in vivo

**DOI:** 10.1186/s12935-020-01372-y

**Published:** 2020-06-29

**Authors:** Syed Umar Faruq Syed Najmuddin, Zahiah Mohamed Amin, Sheau Wei Tan, Swee Keong Yeap, Jeevanathan Kalyanasundram, Muhamad Alhapis Che Ani, Abhimanyu Veerakumarasivam, Soon Choy Chan, Suet Lin Chia, Khatijah Yusoff, Noorjahan Banu Alitheen

**Affiliations:** 1 Institute of Bioscience, Universiti Putra Malaysia, Serdang, Selangor Darul Ehsan 43400 Malaysia; 2 Faculty of Biotechnology and Biomolecular Sciences, Universiti Putra Malaysia, Serdang, Selangor Darul Ehsan 43400 Malaysia; 3 Xiamen University Malaysia, Jalan Sunsuria, Bandar Sunsuria, Sepang, Selangor Darul Ehsan Malaysia; 4 Sunway University, 5, Jalan Universiti, Bandar Sunway, Subang Jaya, 47500 Selangor Darul Ehsan Malaysia; 5 School of Foundation Studies, Perdana University, Block B and D1, MAEPS Building, MARDI Complex, Jalan MAEPS Perdana, 43400 Serdang, Selangor Darul Ehsan Malaysia; 6 Malaysian Genome Institute, National Institute of Biotechnology, Kajang, Jalan Bangi, 43000 Selangor Darul Ehsan Malaysia

**Keywords:** Colon cancer, Recombinant NDV, Apoptosis, Immune systems

## Abstract

**Background:**

Oncolytic viruses have emerged as an alternative therapeutic modality for cancer as they can replicate specifically in tumour cells and induce toxic effects leading to apoptosis. Despite the great potentials and promising results shown in multiple studies, it appears that their efficacy is still moderate and deemed as not sufficient in clinical studies. In addressing this issue, genetic/molecular engineering approach has paved its way to improve the therapeutic efficacy as observed in the case of herpes simplex virus (HSV) expressing granulocyte–macrophage colony-stimulating factor (GM-CSF). This study aimed to explore the cytotoxicity effects of recombinant NDV strain AF2240-i expressing interleukin-12 (rAF-IL12) against CT26 colon cancer cells.

**Methods:**

The cytotoxicity effect of rAF-IL12 against CT26 colon cancer cell line was determined by MTT assay. Based on the IC_50_ value from the anti-proliferative assay, further downward assays such as Annexin V FITC and cell cycle progression were carried out and measured by flow cytometry. Then, the in vivo study was conducted where the rAF-IL12 viral injections were given at the intra-tumoral site of the CT26 tumour-burden mice. At the end of the experiment, serum biochemical, T cell immunophenotyping, serum cytokine, histopathology of tumour and organ section, TUNEL assay, and Nanostring gene expression analysis were performed.

**Results:**

The rAF-IL12 induced apoptosis of CT26 colon cancer cells in vitro as revealed in the Annexin V FITC analysis and also arrested the cancer cells progression at G_1_ phase of the cell cycle analysis. On the other hand, the rAF-IL12 significantly (p < 0.05) inhibited the growth of CT26 tumour in Balb/c mice and had regulated the immune system by increasing the level of CD4 + , CD8 + , IL-2, IL-12, and IFN-γ. Furthermore, the expression level of apoptosis-related genes (bax and p53) was up-regulated as a result of the rAF-IL12 treatment. Additionally, the rAF-IL12 had also down-regulated the expression level of KRAS, BRAF, MAPK1, Notch1, CCL2, and VEGF oncogenes. Besides, rAF-IL12 intra-tumoral delivery was considered safe and not hazardous to the host as evidenced in pathophysiology of the normal tissues and organs of the mice as well as from the serum biochemistry profile of liver and kidney.

**Conclusions:**

These results indicated that rAF-IL12 had better anti-tumoral and cytotoxicity effects compared to its parental wild-type, AF2240-i in combatting the CT26 colon cancer model.

## Background

Newcastle disease virus (NDV) was once a threat to the poultry producers worldwide. It negatively impacts upon egg production, egg weight, and mortality towards avian since its outbreak in 1926s [1, 2]. This single-stranded, non-segmented, negative-sense RNA avian avulavirus can be classified into three pathotypes depending on its virulence: lentogenic (low), mesogenic (medium), and velogenic (high) depending on a mean death time in chicken embryo [3]. Despite causing devastating effects on poultry industry, NDV is known for its pronounced anti-tumoral effect and has the potential to be used as a therapeutic agent in the treatment of cancer. It is a replication-competent oncolytic virus which selectively replicates in cancer cells efficiently and destroys them without compromising the normal cells [4]. Of particular relevance, NDV is also capable in destroying cancer cells that are resistant to certain types of chemotherapy and apoptosis-resistant tumour cells from hypoxic tumour tissue [5, 6]. Various strains of NDV have been shown to kill the cancer cells. For example, the NDV-LaSota strain kills human pancreatic cancer cells, NDV-73-T strain kills cervical carcinoma, fibrosarcoma, and osteosarcoma, and MTH-68/H strain causes significant regression of human tumour cancer cell lines such as HT29, HCT116, and MCF7 [7–9]. It is also important to note that NDV can be tailored to enhance its therapeutic effects towards cancer cells [10]. For example, incorporation of interleukin-2 (IL-2) gene in NDV causes a stable expression of the cytokine, which eventually stimulates the T-cell response hence, increase the viral anti-tumour capacity [11]. In other study, it has been demonstrated that NDV-LaSota strain expressing the rabies virus glycoprotein enhanced the apoptotic induction of lung adenocarcinoma A549 cells [4].

In this present study, the effect of a recombinant NDV strain AF2240-i carrying the interleukin 12 gene (rAF-IL12) on CT26 colon cancer cells was studied in vitro as well as on CT26 colon cancer-bearing mice. AF2240-i is a Malaysian viscerotropic velogenic NDV strain that has been used as standard challenge virus in developing local chicken vaccine [12]. Previously, this strain has been shown to have an oncolytic effect on WEHI-3b leukemia cell line [13] as well as apoptosis-inducing effects on mammary carcinoma cell line [14] while IL-12 cytokine is one of the most powerful anti-tumour cytokines, playing distinct roles in the regulation of anti-tumour immune responses [15]. Therefore, the results of this study would provide an insight into the effect of rAF-IL12 towards colon cancer in vitro and in vivo and may serve as a potential adjunctive treatment for colon cancer.

## Materials and methods

### Preparation of cell culture

Murine colon cancer cell line, CT26 was purchased from The American Type Culture Collection (ATCC), Manassas, VA, USA. The cell line was cultured in Dulbecco’s Modified Eagle Medium (DMEM) medium (Sigma-Aldrich, St. Louis, MO, USA). The medium was supplemented with 10% fetal bovine serum (FBS) (Gibco, Waltham, MA, USA) and 1% penicillin/streptomycin (Gibco, Waltham, MA, USA). The cells were incubated in a humidified incubator at 37 °C in the presence of 5% CO_2_ and were subcultured upon reaching 70% confluency.

### Preparation of virus

The parental Malaysian viscerotropic NDV strain, AF2240-i was used as positive control throughout this study. The rAF-IL12 virus was developed in the Virology Laboratory, Faculty of Biotechnology and Biomolecular Sciences, Universiti Putra Malaysia [16]. The viruses were propagated in allantoic fluid of 9–11 days-old SPF embryonated chicken eggs and were incubated at 37 °C for 48–72 h. The allantoic fluid was harvested and the titre of the virus was determined by the haemagglutinin assay (HA) using 1% chicken red blood cells.

### Grouping for in vitro studies

**Table:** 

Group	Explanation
1. Control	‘Negative control of the study’ whereby no treatment was given
2. AF2240-i	AF2240-i viral treatment and act as the ‘positive control of the study’ which will be compared to the other viral treatment (i.e. rAF-IL12)
3. rAF-IL12	‘Main subject of interest’ for this study which will be compared to the AF2240-i (positive control) treatment

### In vitro 3- [4,5-dimethylthiazol-2-yl]-2,5 diphenyltetrazolium bromide (MTT) assay

Briefly, a concentration of 8000 cells/well were seeded into a 96-well plate and incubated overnight before being infected with a serial dilution of the virus on the following day as described by Kumar et al. [17]. After 72 h of treatment, MTT reagent solution (5 mg/mL in phosphate-buffered saline (PBS)) (Promega, Madison, WI, USA) was added into each well and incubated for 2–3 h. Later, the solutions were removed, and the formazan crystals formed were solubilized with dimethyl sulfoxide, DMSO. The plate was read using a μQuant™ enzyme-linked immunosorbent assay (ELISA) plate reader (Bio-Tek Instruments, Winooski, VT, USA) at a 570 nm wavelength. The percentage of cell viability was calculated by the given formula:

$$ {\text{\%  of cell viability}}  =  {\text{[Absorbance at }} \times {\text{ concentration (virus sample)] / [Absorbance at 0 concentration (control)] }} \times {\text{ 100}} $$

### qPCR validation of virus copy number

The CT26 cells were cultured in 6-well plates at a concentration of 2 × 10^5^ cells/well and incubated overnight. On the following day, virus treatment (AF2240-i and rAF-IL12) was added and incubated for 72 h. Upon reaching the time-point, viral RNA was extracted from the treatment samples using TRI Reagent^®^ (Sigma-Aldrich, St. Louis, MO, USA) and the yield and purity of RNA were then assessed using Nanodrop machine (Eppendorf, Hauppauge, NY, USA) before qPCR validation of virus copy number was performed. A Taqman real-time PCR protocol was carried out using a published forward primer (5′ TCC GCA AGATCC AAG GGT CT 3′), reverse primer (5′ CGC TGTTGC AAC CCC AAG 3′) and TaqMan probe (5′ (FAM) AA GCG TTT CTG TCT CCT TCC TCC A (BHQ) 3′) that target the fusion (F) gene of the NDV [18]. All qPCR reactions were performed in a final volume of 20 µL reaction mixture containing 1X of iTaq universal probes reaction mix (Bio-Rad Laboratories Inc., Hercules, CA, USA), 0.5 µM of each forward and reverse primer, 0.25 µM of TaqMan probe, 1 unit of iScript reverse transcriptase, and 300 ng of RNA as outlined by Abdolmaleki et al. [18]. No template control was included in each run. RNA conversion to cDNA, cDNA amplification, and quantification was performed using a Bio-Rad CFX96™ Real-Time System PCR machine (Bio-Rad Laboratories Inc., Hercules, CA, USA). Data was collected at the end of run and data analysis was performed using the CFX Manager™ Software version 1.6 (Bio-Rad Laboratories Inc., Hercules, CA, USA).

### In vitro Annexin V FITC analysis

The assay was carried out using Annexin V FITC Kit (BD Pharmigen, Franklin Lakes, NJ, USA) according to manufacturer’s protocol to evaluate the apoptosis induction of rAF-IL12 towards cancer cells. Briefly, the seeded cells (2 x 10^5^ cells/well) were infected for 72 h before the cell pellets were harvested and resuspended in the 1X Binding buffer prior to staining with FITC Annexin V and propidium iodide (PI) dyes as described by Najmuddin et al. [19]. The cell suspension was then allowed to stand in the dark at room temperature for 15 min before being analysed by NovoCyte Flow Cytometer (ACEA Biosciences Inc., San Diego, CA, USA) using NovoExpress^®^ version 1.2.4 software (ACEA Biosciences Inc., San Diego, CA, USA).

### In vitro cell cycle analysis

The CT26 cancer cells were seeded in 6-well plates at a concentration of 2x10^5^ cells/well. After 72 h of treatment, the cells were centrifuged at 2500 x g speed for 5 min to obtain the cells pellet. The cells pellet was then, subjected to cell cycle analysis using Cycletest^TM^ Plus DNA Reagent Kit (BD Biosciences, Franklin Lakes, NJ, USA), as described by Najmuddin et al. [19]. The cells pellet was resuspended in the solution A (trypsin) and incubated for 10 min before solution B (RNAse A) was added. At this stage, a further 10 min was required. Finally, solution C (propidium iodide (PI) stain) was added to the mixture. After 10 min of incubation, the mixture was analysed by NovoCyte Flow Cytometer (ACEA Biosciences Inc., San Diego, CA, USA) using NovoExpress^®^ version 1.2.4 software (ACEA Biosciences Inc., San Diego, CA, USA).

### In vivo animal study

About 5-week old female Balb/c mice, weighing approximately 20 grams were purchased from the animal house, UPM Animal Resource Unit. The mice were accommodated under standard condition as outlined by the UPM ethics committee’s guidelines for the care of laboratory animals. All the animals were fully conducted in humane and ethical manner and were housed under 12-h dark–light cycle with ambient temperature regulated ~ 25 ± 2 °C. Clean tap-water and standard pellets-diet were provided throughout the study period. This study was approved by the International Animal Care and Use Committee (IACUC), UPM (Reference Number: UPM/IACUC/AUP/RO61/2017) and the experimentation was conducted based on the approved guidelines.

#### (i) Injection of normal Balb/c mice with AF2240-i and rAF-IL12 for in vivo pilot study (for viral replication kinetics)

Mice were housed into eight open cages (six mice per cage) and were treated with either AF2240-i or rAF-IL12. In this experimental setup, the mice were given intra-peritoneally shot once (0.1 mL of 2^7^ HA titre of AF2240-i or rAF-IL12 virus) and sacrificed upon reaching their time-point (day-2, day-3, day-7, or day-10).

#### (ii) Injection of tumour-challenged Balb/c mice with AF2240-i and rAF-IL12 for in vivo pilot study (for viral replication kinetics)

Mice were housed into six open cages (six mice per cage) and were given intra-tumoral shot once (0.1 mL of 2^7^ HA titre of AF2240-i or rAF-IL12 virus) and sacrificed upon reaching their time-point (day-2, day-3, or day-7).

#### (iii) Viral injection of tumour-challenged Balb/c mice to determine anti-tumoral effects of AF2240-i and rAF-IL12 (in vivo complete study)

Mice were housed into four open cages, which specifically defines their respective groups; normal, negative control, AF2240 virus-treated, and rAF-IL12-treated. Normal mice group indicated that the mice were not bearing any tumour and no treatment was given. On the other hand, negative control group indicated that the tumour-bearing mice were treated with phosphate-buffered saline (PBS). All the mice in the treatment groups were given intra-tumoral shot (0.1 mL of 2^7^ HA titre dosage for AF2240-i and rAF-IL12 or 0.1 mL of PBS for PBS-treated group) twice a week for four cycles (i.e.; 8 times in 28 days-treatment).

**Table:** 

Group	Explanation
1. Normal	Healthy mice which were not bearing any tumor during this study. No treatment was given to the mice
2. Negative control	‘Negative control of the study’ whereby phosphate-buffered saline (PBS) was given to the tumor-burden mice
3. AF2240-i	AF2240-i viral treatment given to tumor-burden mice and act as the ‘positive control of the study’ which will be compared to the other viral treatment (i.e. rAF-IL12)
4. rAF-IL12	‘Main subject of interest’ for this study which will be compared to the AF2240-i (positive control) treatment. The rAF-IL12 viral treatment was given to the tumor-burden mice

### Cancer cell preparation and injection into mice

The CT26 murine colon carcinoma cell line was used in the latter two studies and was harvested from tissue culture flask at 70% cell confluency. The cell suspension was prepared in PBS to have approximately 10^6^ cells/mL for injection. Later, cancer cells inoculation (100 µL) was made at the subcutaneous site of the right hind leg of the mice using a 27-gauge needle (Terumo Corporation, Tokyo, Japan). The mice were observed daily for about 5 days until the tumour masses develop (± 50 mm^3^).

### Measurement of tumour growth

Tumours were measured with a vernier caliper once a week. Tumours growth were monitored and plotted against time after treatment was given. Tumour size was measured using the standard formula as described by Kersemans et al. [20]:

$${\text{Tumour volume (mm}}^{3} )  =  0.5 \times {\text{ length }} \times {\text{ (width)}}^{2}.  $$

### Tissue collection

Upon reaching the time-point at day 29, the mice were sacrificed by cervical dislocation in accordance with IACUC guidelines prior to sample collection. Tissue samples such as tumours and vital organs (lung, spleen, liver, and kidney) were harvested. One half of the tissues were placed in tube containing 10% buffered formalin for fixation and histopathology analysis while the other half were placed in RNAlater solution (ThermoFisher Scientific, Waltham, MA, USA) and stored in −80 °C freezer for molecular analysis.

### Hematoxylin and eosin (H&E) histopathology staining

The H & E histopathology staining was carried out according to the protocol described by Cardiff et al. [21]. This assay involved the deparaffinized step of tissue sections in xylene for 5 min before the rehydration step was carried out by immersing the slides into 100, 90, 80 and 70% ethanol solution. Later, the slides were stained with Harris Hematoxylin (Sigma-Aldrich, St. Louis, MO, USA) for 5 min and counterstained with eosin for 30 secs and followed by a sequential dehydration step in the 60–100% ethanol solution for 10 secs before clearing the slides with xylene. The slides were then, mounted with the DPX mounting media (Sigma-Aldrich, St. Louis, MO, USA) before it can be observed under a bright-field microscope (Nikon, Tokyo, Japan).

### Serum biochemical analysis

Serum biochemical profile was conducted to determine liver and kidney function enzymes and biomarker activities as described by Abu et al. [22]. Briefly, blood was withdrawn from the mice and then subjected to a centrifugation step (10,250x*g* for 5 min) to obtain the serum. Next, the serum was analysed for its level of biochemical properties such as aspartate aminotransferase (AST), alkaline phosphates (ALP), alanine aminotransferase (ALT), and creatinine using standard assay kits (Roche Diagnostics, Indianapolis, IN, USA) by 902 Hitachi automatic analyzer (Hitachi LTD, Tokyo, Japan).

### Immunophenotyping of splenocytes

Immunophenotyping assay was carried out based on the protocol described by Yeap et al. [23]. Spleens obtained from mice were washed with PBS and meshed through a 70 μm cell strainer with a rubber syringe plunger before centrifuged at 2, 500x*g* for 5 min. The red blood cells were then lysed and removed from the cells pellet by incubating in lysis buffer for 10 min at 4 °C. Around 1 x 10^6^ cells were then stained with appropriate fluorochrome-conjugated antibodies (CD3, CD4, and CD8) (Abcam Inc., Eugene, OR, USA) for 2 h on an orbital shaker. Later, the cells were washed with PBS and fixed in 4% paraformaldehyde. The stained cells were then analysed with the NovoCyte Flow Cytometer (ACEA Biosciences Inc., San Diego, CA, USA) and analysis was done using NovoExpress^®^ version 1.2.4 software (ACEA Biosciences Inc., San Diego, CA, USA).

### Serum detection of IL-2, IL-12, and IFN-γ cytokines

The level of cytokine (IL-2, IL-12, and IFN-γ) expression of the treated mice in comparison to normal mice were measured using enzyme-linked immunosorbent assay kits (Biolegend, San Diego, CA, USA) according to the user’s protocol provided. Briefly, the 96-well plates were coated with the designated capture anti-bodies (diluted) followed by the addition of 0.5% bovine serum albumin (BSA), samples (serum), diluted biotin-labelled detection antibody, 1X avidin-horseradish peroxidase (avidin-HRP) conjugate, and 3,3′,5,5′- tetramethylbenzidine (TMB) substrate solution. Finally, the stop solution was added to each well at 1:1 ratio before the absorbance reading was measured at 450 nm and 570 nm wavelengths with µquant microplate reader (Bio-Tek Instruments, Winooski, VT, USA).

### NanoString gene expression analysis

The gene expression analysis of tumour RNA was conducted according to nanoString nCounter TagSet Elements^TM^ user’s guide (NanoString Technologies Inc., Seattle, WA, USA). The sample cartridge was transferred to the Digital Analyzer for imaging and analysis and the results were available for download directly from the Digital Analyzer.

### In vivo Terminal deoxynucleotide transferase (TdT) dUTP Nick-End Labeling (TUNEL) assay

TUNEL assay was conducted using the DeadEnd^TM^ colorimetric TUNEL System (Promega, Madison, WI, USA) according to the manufacturer’s protocol [24]. The slides were viewed under bright-field inverted microscope (Nikon, Tokyo, Japan).

### Statistical analysis

All experiments were evaluated by one-way ANOVA using GraphPad Prism 7 software (GraphPad Software Inc., San Diego, CA, USA). All in vitro and in vivo tests were carried out by three and six replicates, respectively and the results are expressed as mean ± standard error of mean (S.E.M.). Values p < 0.05 were considered as statistically significant.

## Results

### Viral replication kinetics in CT26 cells

**Fig. 1:**
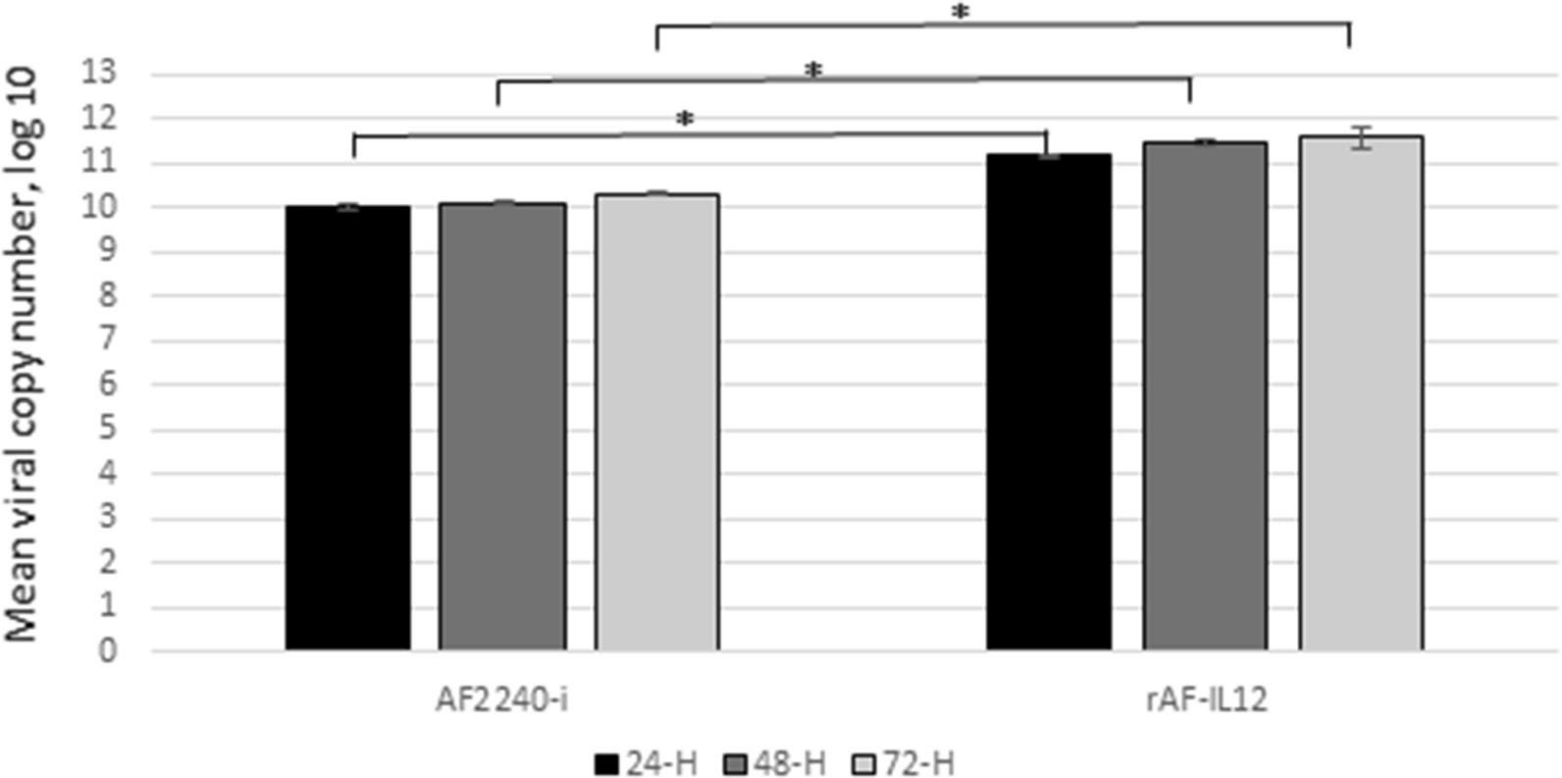
Viral copy number of AF2240-i and rAF-IL12 detected in CT26 cells at 24, 48, and 72 h post-infection as measured by RT-qPCR analysis based on NDV Fusion (F) gene for viral replication kinetics. The copy number was calculated based on the formula generated from the qPCR standard curve of the NDV: X = (y−58.149)/−3.371; where X is the viral copy number; y is the value mean Cq; 58.149 is the y-intercept value; and −3.371 is the slope of the standard curve. Data are presented as mean ± S.E.M from triplicate determinations. Statistically significant differences between the means were determined by One-Way ANOVA followed by Duncan post hoc test. Differences were considered significant when the ^*^p ≤ 0.05

As shown in Fig. 1, rAF-IL12 managed to replicate in CT26 cells comparable to the parental NDV, AF2240-i throughout the three time-points (24-, 48-, and 72 h). Notably, there was a trend of increasing viral copy number of AF2240-i and rAF-IL12 from the 24-h to 72-h post-infection.

### In Vitro Cytotoxicity Effects of rAF-IL12 in CT26 Cells

**Fig. 2:**
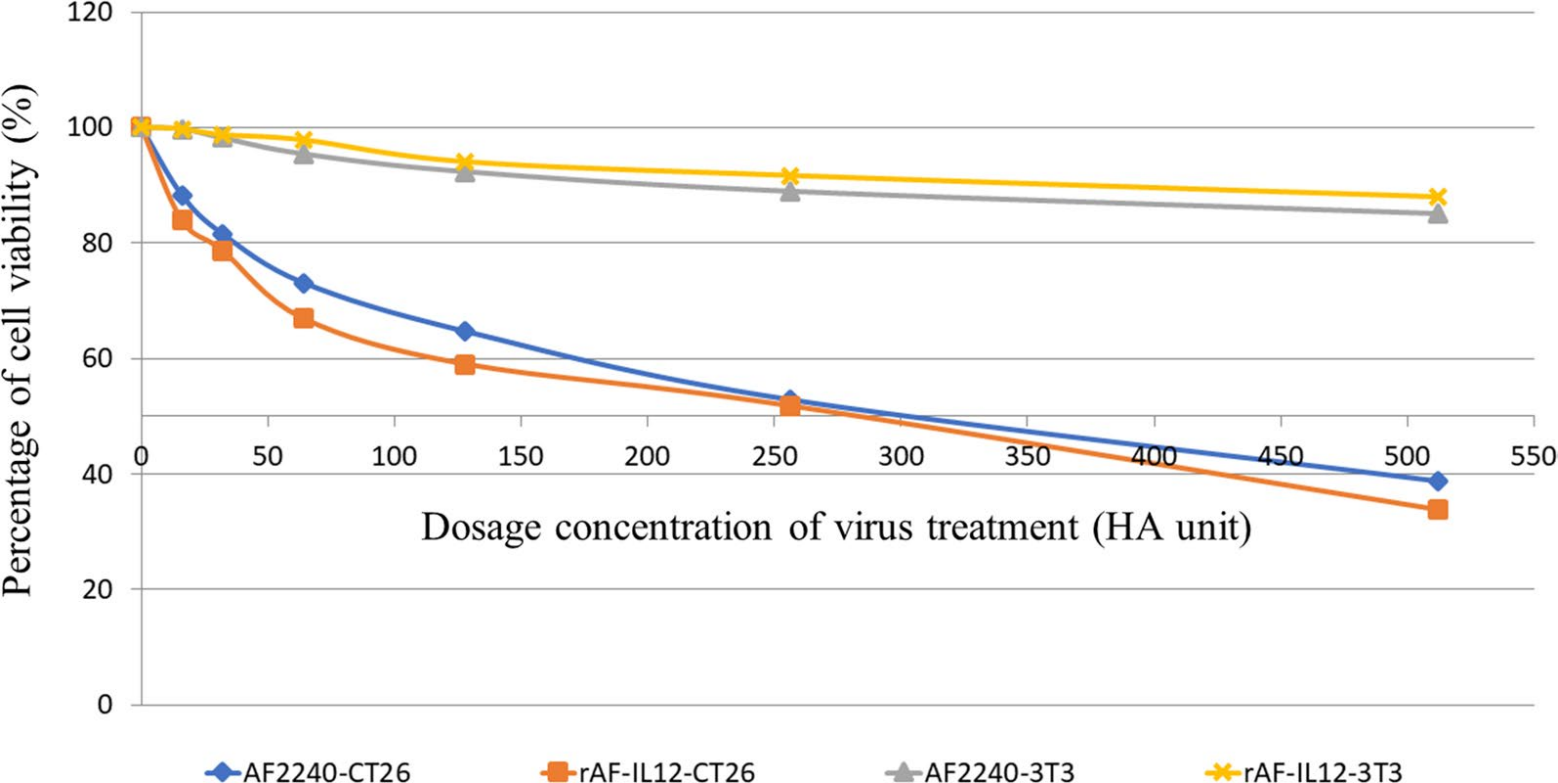
MTT assay showing the cytotoxicity activity of AF2240-i and rAF-IL12 against CT26 and 3T3 cells 72 h post-infection. Percentage of viability of two cell lines (CT26 and 3T3) when treated with seven doses of virus treatment (AF2240-i or rAF-IL12) after 72 h. The IC_50_ value (half-maximal inhibitory concentration, HA unit) for AF2240-i-CT26 = 291 ± 0.67; rAF-IL12-CT26 = 276 ± 0.88; AF2240-3T3 = n.d.; rAF-IL12-3T3 = n.d. Data are presented as mean ± S.E.M from triplicate determinations

**Fig. 3:**
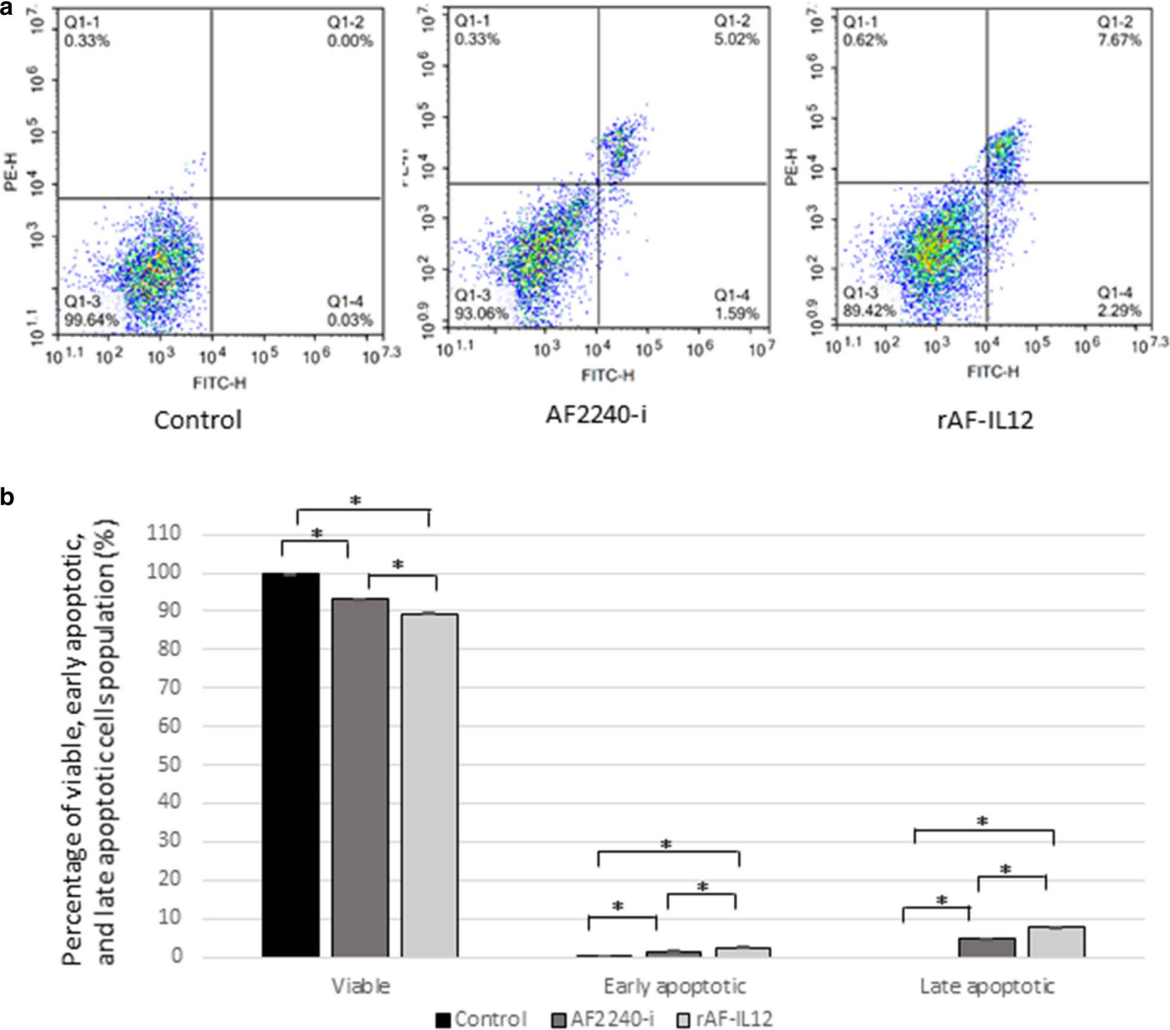
Annexin V/FITC assay in CT26 cells following AF2240-i (291 HA titre) and rAF-IL12 (276 HA titre) 72 h post-infection. **a** Typical quadrant analysis of Annexin V/FITC flow cytometry of CT26 cells apoptosis. The lower left quadrant of each group indicated the viable cells population; the lower right quadrant indicated the early apoptotic cells population; the upper right quadrant indicated the late apoptotic cells population; and the upper left quadrant indicated the necrotic cells population. Two fluorescent dyes were used in this assay which are FITC (x-axis) and PE (y-axis). **b** Percentage of viable, early apoptotic, and late apoptotic cells population analysed by quantitative analysis. Data are presented as mean ± S.E.M from triplicate determinations. Mean values with statistical difference at p < 0.05 between control, AF2240-i, and rAF-IL12 are indicated with *

**Fig. 4:**
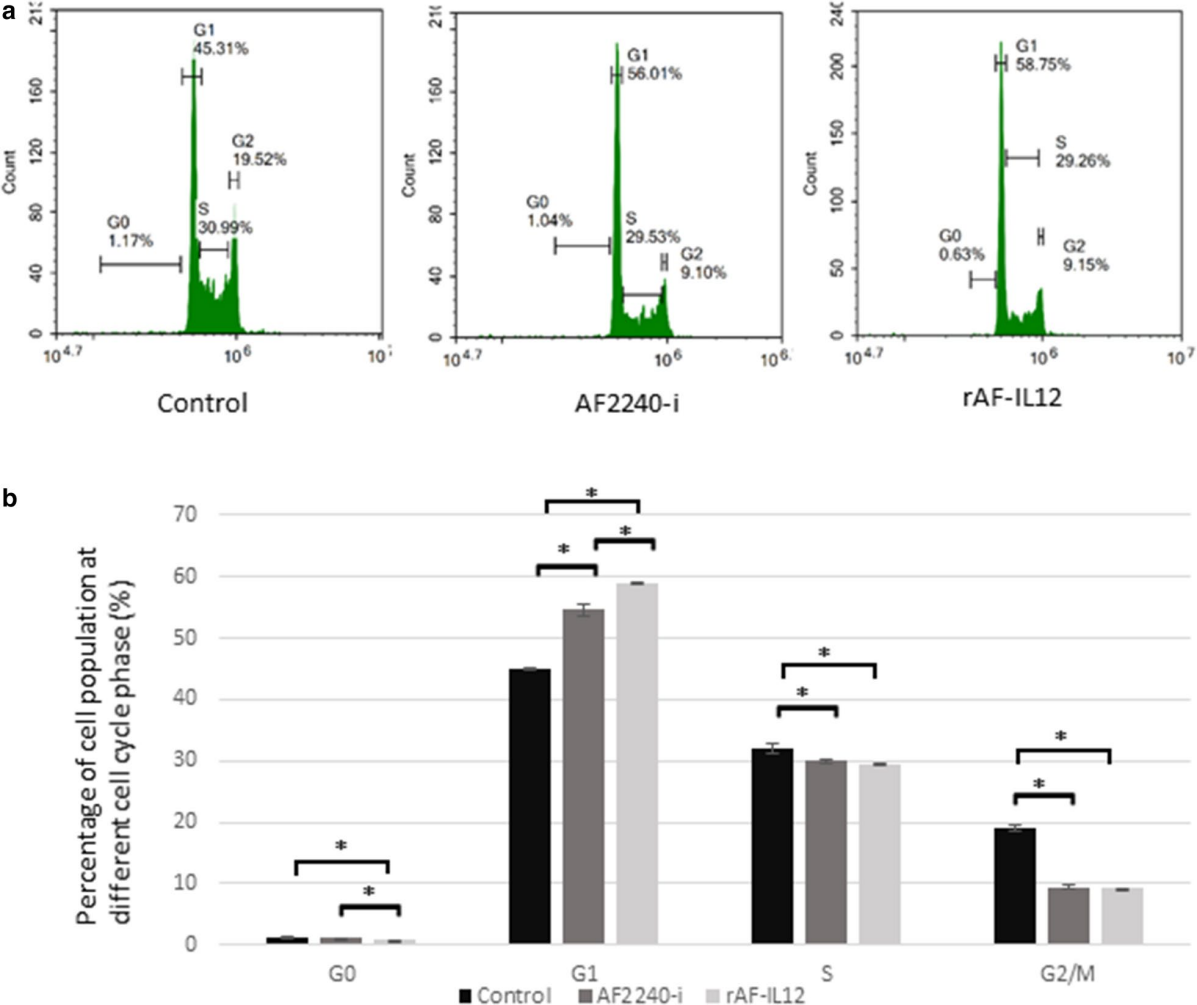
Cell cycle analysis of CT2 cells at different cell cycle phase. **a** Histogram of cell cycle analysis showing distribution of CT26 cells at different cell cycle phase (G_0_, G_1_, S, and G_2_) after 72 h period of treatment with AF2240-i and rAF-IL12. The G_0_ peak appeared first in the histogram followed by G_1_, S, and G_2_ peaks indicating the percentage of cells population in those aforementioned cell cycle phases. Percentage of cells population in each peak was calculated from a total number of 10, 000 cells in each flow cytometry run. **b** Percentage of cells population at different cell cycle phase analysed by flow cytometer following treatment with AF2240-i (291 HA titre) and rAF-IL12 (276 HA titre) in CT26 cells. Data are presented as mean ± S.E.M from triplicate determinations. Mean values with statistical difference at p < 0.05 between control, AF2240-i, and rAF-IL12 are indicated with *

Cytotoxic effects of rAF-IL12 against CT26 colon cancer cell line in comparison to the parental NDV, AF2240-i was assessed by MTT assay (Fig. 2). It is noted that rAF-IL12 inhibited CT26 cancer cells proliferation at a lower dose than the parental NDV, AF2240-i based on IC_50_ (half-maximal inhibitory concentration) value of rAF-IL12 (276 ± 0.88 HA unit) and AF2240-i (291 ± 0.67 HA unit) as shown in Table 1. However, both viruses did not inhibit the proliferation of normal fibroblast 3T3 cells. The role of rAF-IL12 in causing cancer cell death or apoptosis was evaluated by the Annexin V/FITC assay. Figure 3 shows the results of Annexin V/FITC assay of CT26 cells after infected with AF2240-i and rAF-IL12 viruses for 72 h. The percentage of early apoptotic cells increased from 0.03% ± 0.01% in the control group to 1.44% ± 0.12% in AF2240-i-treated cells and 2.5% ± 0.16% in rAF-IL12-treated cells (Fig. 3b). The percentage of late apoptotic cells was even higher in both AF2240-i- and rAF-IL12-treated cells (4.92% ± 0.08% and 7.55% ± 0.11%, respectively) whereas none was detected in the control group. Meanwhile, cell cycle analysis by flow cytometry was performed to evaluate the effects of rAF-IL12 treatment on the CT26 cell cycle progression (Fig. 4). From Fig. 4b, there was a significant increase in the percentage of CT26 cells at the G_1_ checkpoint in AF2240-i (54.58% ± 1.02%) and rAF-IL12 (58.85% ± 0.11%) groups compared to control group (45.04% ± 0.21%). The cells percentage at G_2_ checkpoint had a substantial decrease from 19.01% ± 0.42% in the control group to 9.44% ± 0.35% and 9.19% ± 0.03% in AF2240-i and rAF-IL12 groups, respectively.

**Table 1:** IC_50_ (half-maximal inhibitory concentration, HA unit) of AF2240-i and rAF-IL12 in CT26 and 3T3 cells post 72 h post-infection

Cell line	Virus treatment	IC_50_ (half-maximal inhibitory concentration, HA unit)
CT26	AF2240-i	291 ± 0.67
	rAF-IL12	276 ± 0.88^*^
3T3	AF2240-i	N/A
	rAF-IL12	N/A

Data are presented as mean ± S.E.M from triplicate determinations. Statistically significant differences between the means at p < 0.05 are indicated with *

### rAF-IL12 treatment impeded the growth of tumour in vivo

**Fig. 5:**
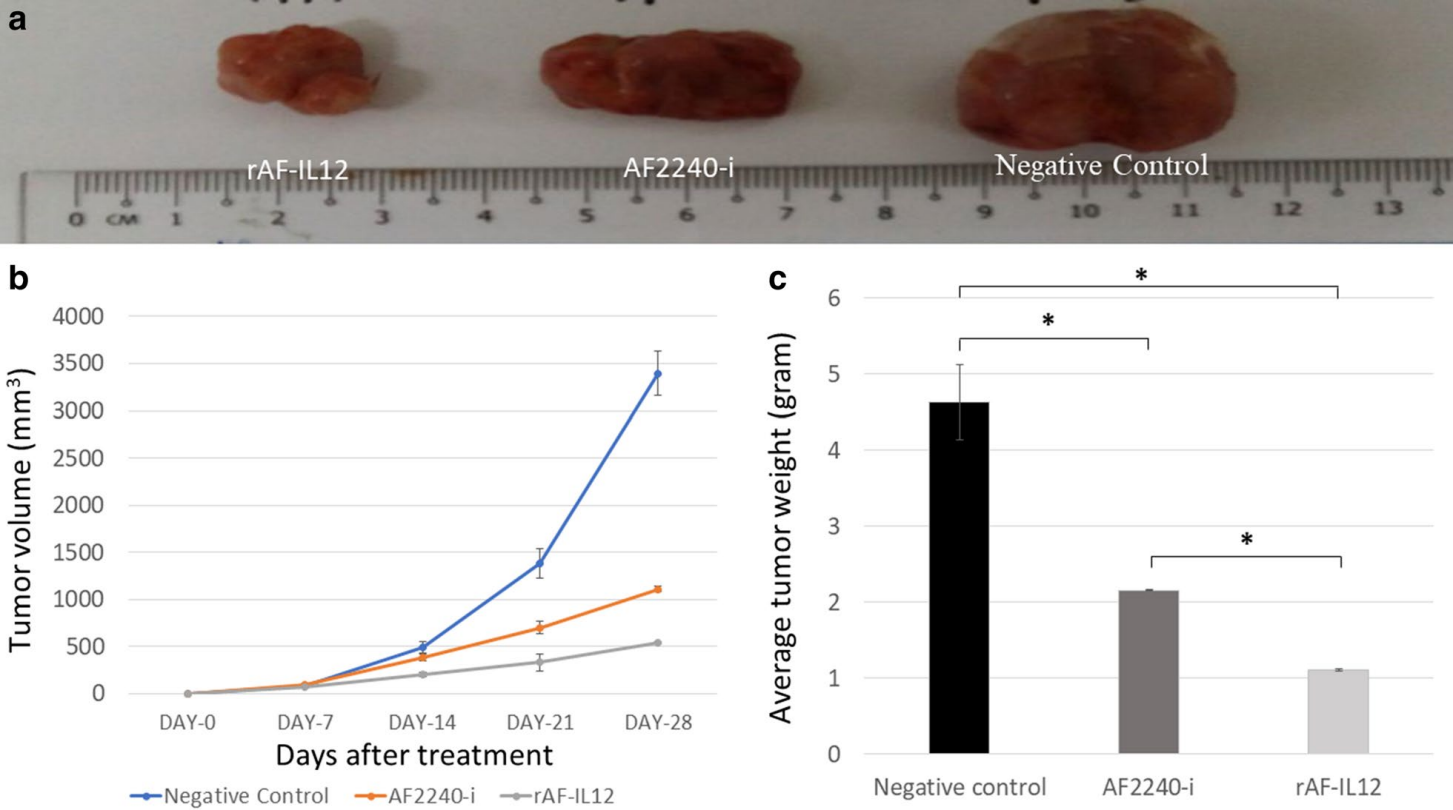
Effects on the CT26 tumours for the negative control, AF2240-i, and rAF-IL12 groups. **a** Images of CT26 tumour harvested from negative control, AF2240-i, and rAF-IL12 groups following the 28-days of treatment. **b** The growth rate of the CT26 tumours from day-0 until day-28 of treatments. **c** Average weight of CT26 tumours harvested from mice after 28-days of treatment. Data are represented as mean ± S.E.M. of six mice per group. Mean values with statistical difference at p < 0.05 between control, AF2240-i, and rAF-IL12 are indicated with *

The rAF-IL12 treatment group had visibly the smallest size of CT26 tumour among the other groups as shown in Fig. 5a. Tumours in the negative control group had vigorous growth and proliferation from day-0 to day-28 as opposed to tumours receiving treatments with rAF-IL12 and AF2240-i (i.e. at a slower growth rate) as shown in Fig. 5b. At day-28, the mice that were treated with rAF-IL12 had significantly (p < 0.05) smaller tumour volume (537.90 ± 12.99 mm^3^) compared to AF2240-i treated group (1113.00 ± 32.16 mm^3^) and the negative control group (3398 ± 229.80 mm^3^). Moreover, rAF-IL12 had significantly (p < 0.05) the lowest mean tumour weight (1.11 ± 0.36 g) compared to the negative control (4.63 ± 0.77 g) and AF2240-i treated group (2.16 ± 0.68 g) (Fig. 5c).

### rAF-IL12 increased the number of apoptotic cells and reduced the number of mitotic cells

**Fig. 6:**
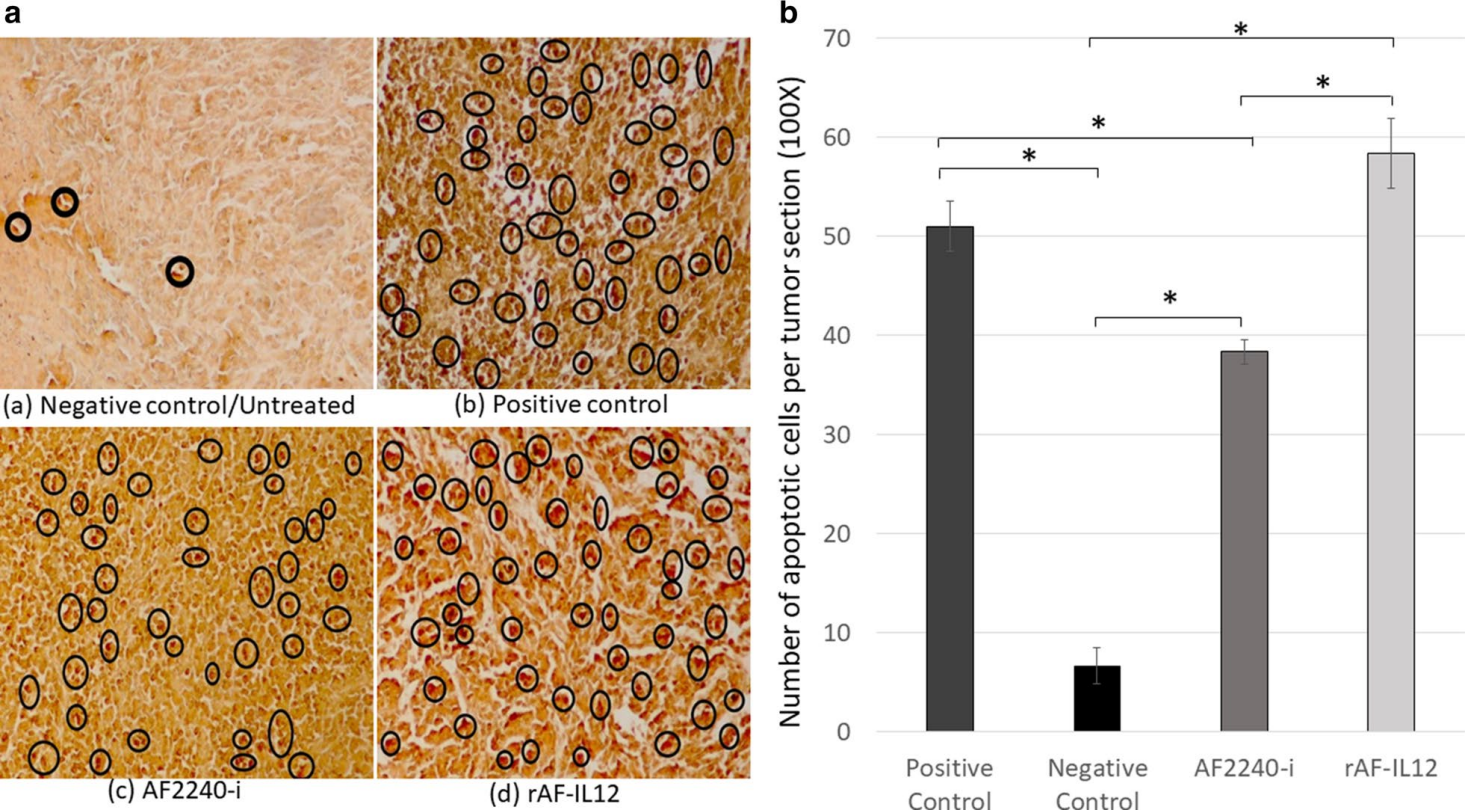
**a** Tumour sections assayed by DeadEnd colorimetric TUNEL system to indicate cell apoptosis in CT26 tumour-bearing Balb/c mice: **a** positive control (sample treated with DNAse I); **b** negative control; **c** tumour treated with AF2240-i; **d** tumour treated with rAF-IL12. Brown stained nuclei in the black circle indicate DNA fragmentation and nuclear condensation. Magnification: 100x; **b** The number of apoptotic cells per tumour section from the four aforementioned groups after 28 days of treatment. A total number of 200 cells per tumor section was used for the quantification analysis in the TUNEL assay. Data are presented as mean ± S.E.M. of six mice per group. Mean values with statistical difference at p ≤ 0.05 between groups are indicated with *

**Fig. 7:**
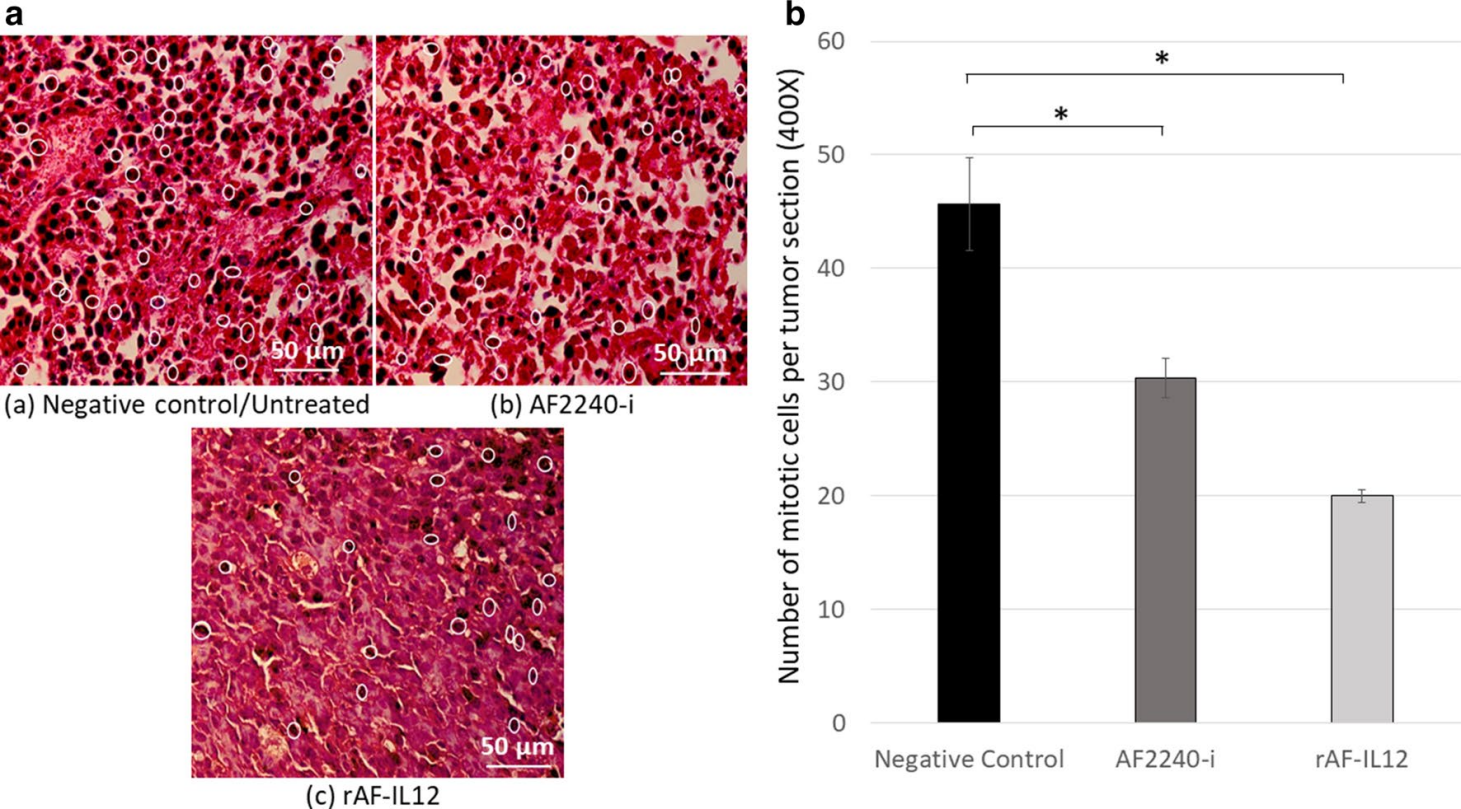
**A** Photomicrograph section of the tumour mass of mice stained with H&E from three different groups of mice, **a** Untreated, **b** AF2240-i-treated, and **c** rAF-IL12-treated. The white circles indicate the actively dividing/mitotic tumour cells in the aforementioned groups. Magnification: 400X; H&E scale bar = 50 µm; **B** The number of mitotic cells per tumour section from the negative control, AF2240-i-treated, and rAF-IL12-treated after 28-days of treatment. Data are presented as mean ± S.E.M. of six mice per group. Mean values with statistical difference at p ≤ 0.05 between groups are indicated with *

In situ chromatin fragmentation was confirmed by TUNEL staining of the sectioned tumours of negative control, positive control, AF2240-i, and rAF-IL12 groups as shown in Fig. 6a. Based on Fig. 6b, TUNEL-stained sections of rAF-IL12 treated group had significantly (p < 0.05) highest number of dark brown apoptotic nuclei in the tumour (58.33 ± 3.53) compared to the negative control and AF2240-i treated groups (6.67 ± 1.86 and 38.33 ± 1.20, respectively). In addition, there was no significance difference (p > 0.05) in the number of apoptotic nuclei between the rAF-IL12-treated group and positive control (i.e. sectioned tumour treated with DNAse I). Meanwhile, Fig. 7a showed the histological assessment of the H&E stained tumour mass of the negative control, AF2240-i, and rAF-IL12 groups. Based on Fig. 7b, treatment with AF2240-i and rAF-IL12 had significantly (p < 0.05) decreased the number of actively dividing/mitotic cells when compared to the negative control group.

### rAF-IL12 regulated the immune response in immunocompetent Balb/c mice

**Fig. 8:**
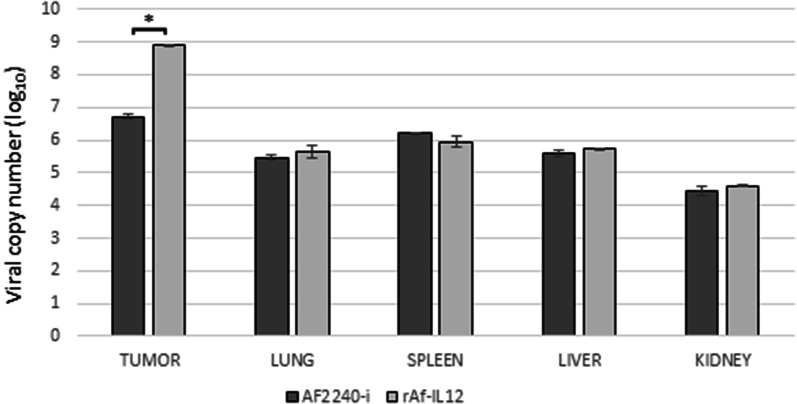
Percentage of **a** CD4 +/CD3 + and (B) CD8 +/CD3 + T-cell population from spleenocytes of normal, negative control, AF2240-i, and rAF-IL12 groups after 28 days of treatment as analysed by flow cytometry. Data are presented as mean ± S.E.M. of six mice per group. Mean values with statistical difference at p ≤ 0.05 between groups are indicated with *

As shown in Fig. 8a, the negative control mice showed a significant (p < 0.05) reduction in percentage of CD4 +/CD3 + T-cell population when compared to the normal mice group (22.66% and 11.03%, respectively). Treatment with AF2240-i and rAF-IL12 significantly (p < 0.05) increased the CD4 +/CD3 + T-cell population to 12.76% and 18.5%, respectively. A similar pattern was observed in Fig. 8b as the negative control mice had a significant (p < 0.05) decrease in the percentage of CD8 +/CD3 + T-cell population (4.69%) when compared to the normal mice group (9.02%). Meanwhile, the CD8 +/CD3 + T-cell population was significantly (p < 0.05) increased in the AF2240-i and rAF-IL12-treated groups (5.20% and 7.86%, respectively). These results showed that rAF-IL12 could significantly (p < 0.05) upregulate higher level of expression of CD4 +/CD3 + and CD8 +/CD3 + T-cell populations when compared to the parental NDV, AF2240-i.

**Fig. 9:**
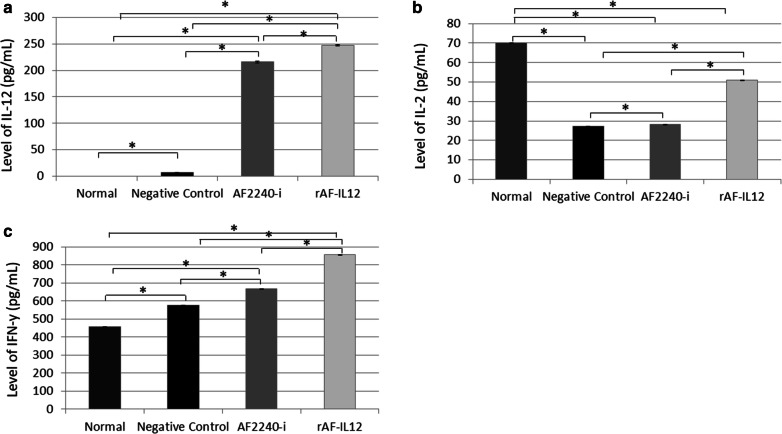
Serum level of cytokine **a** interleukin-12, **b** interleukin-2, and **c** interferon-γ from normal and colon cancer-challenged mice (negative control, AF2240-i, and rAF-IL12) after 28-days of treatment. Data are presented as mean ± S.E.M. of six mice per group. Mean values with statistical difference at p ≤ 0.05 between groups are indicated with *

According to Fig. 9 (a–c), serum cytokine IL-12, IL-2, and IFN-γ were increased significantly (p < 0.05) in mice receiving rAF-IL12 treatment when compared to either negative control or AF2240-i treated mice. An increasing pattern of cytokines level (IL-12 and IFN-γ) was also observed shifting from normal to negative control, AF2240-i, and rAF-IL12 treated mice (Fig. 9a–c). The mice treated with AF2240-i and rAF-IL12 showed a significant (p < 0.05) increase in the expression level of IL-2 (28.15 pg/mL and 50.81 pg/mL, respectively) when compared to the negative control group (27.1 pg/mL) (Fig. 9b). Nevertheless, the normal mice group showed the highest IL-2 concentration (70.08 pg/mL) amongst the others.

### rAF-IL12 modulated the expression of genes related to the apoptosis, metastasis, and angiogenesis

**Fig. 10:**
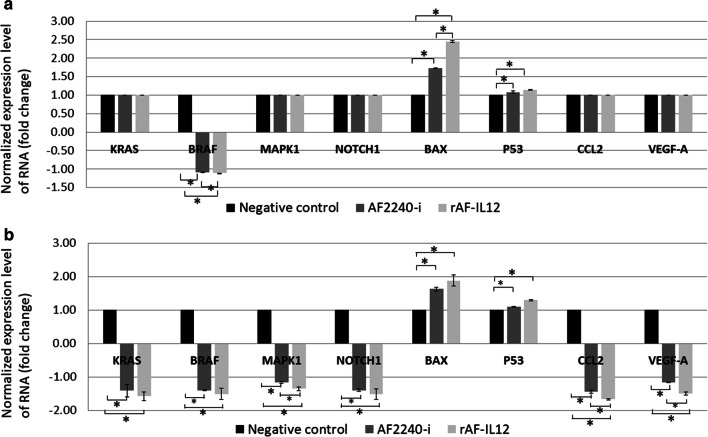
Normalized gene expression level of KRAS, BRAF, MAPK1, NOTCH1, BAX, p53, CCL2, and VEGF-A in **a** CT26 cancer cells in vitro and **b** tumour excised from CT26 tumour-burden mice. Data are presented as mean ± S.E.M from three independent experiments. Statistically significant differences between the means were determined by One-Way ANOVA followed by Duncan post hoc test. Differences were considered significant when the p ≤ 0.05 as indicated by ^*^

The expression level of several genes related to apoptosis mechanism, metastasis, and angiogenesis were measured by nanoString nCounter TagSet Elements^TM^ gene expression analysis. Based on Fig. 10a, in vitro treatment of rAF-IL12 had significantly (p < 0.05) decreased BRAF gene expression and significantly (p < 0.05) increased the expression level of BCL2 associated X (BAX) and p53 while there was no difference in expression level of other genes. Meanwhile, rAF-IL12 treatment in vivo significantly (p < 0.05) decreased the expression level of Kirsten rat sarcoma (KRAS), BRAF, mitogen-activated protein kinase 1 (MAPK1), NOTCH1, C–C motif chemokine ligand 2 (CCL2), and vascular endothelial growth factor-A (VEGF-A) in the tumour and significantly (p < 0.05) increased the expression level of BAX and p53 (Fig. 10b).

### rAF-IL12 replicated in the tumour and disseminated into vital organs

Additional file 1: Figure S1A showed that the AF2240-i viral copy number in the lung, spleen, liver, and kidney of the normal Balb/c mice reached the highest peak at day-2 post-infection and started to decrease at day-3 until day-10. A similar pattern was also observed for Additional file 1: Figure S1B. In the CT26 tumour-challenged mice study, the AF2240-i viral copy number reached the highest peak at day-2 post-infection in the tumour and lung; reached the highest peak at day-3 post-infection in the liver and kidney; and reached the highest peak at day-7 post-infection in the spleen as shown in Additional file 2: Figure S2A. Meanwhile, Additional file 2: Figure S2B showed that the rAF-IL12 viral copy number of the tumour had the highest peak at day-7 post-infection; had the highest peak at day-2 post-infection in the lung and kidney; and had the highest peak at day-3 post-infection in the liver and spleen. Additional file 3: Figure S3 showed that the rAF-IL12-treated mice had significantly (p < 0.05) higher viral copy number in tumour (8.89 (log10)) compared to the AF2240-i-treated mice (6.72 (log10)) at the end (i.e. day-28) of the anti-tumoral effects study. On the other hand, there were no significant difference in the level of viral copy number between the AF2240-i and rAF-IL12 treated groups in the lung, spleen, liver, and kidney.

### rAF-IL12 did not interfere with the serum biochemistry profile of the challenged mice

Table 2 shows the level of serum biochemistry for ALP, AST, ALT, and creatinine in four mice groups (normal, negative control, AF2240-i, and rAF-IL12) at the end of the study (day-28). The level of serum ALP, AST, ALT, and creatinine in the negative control group increased significantly (p < 0.05) when compared to serum level in the normal group. Treatment with rAF-IL12 however, had significantly (p < 0.05) decreased to approximately normal serum level of ALP, AST, and ALT (exception to creatinine). These data suggest that rAF-IL12 did not affect the liver and kidney functions based on observations on the production of the serum levels.

**Table 2:** ALP, AST, ALT and creatinine serum biochemistry profiles of normal, negative control, AF2240-i-, and rAF-IL12-treated colon cancer-challenged mice after 28-days of treatments

	ALP (U/L)	AST (U/L)	ALT (U/L)	Creatinine (Umol/L)
Normal	188.70 ± 7.50	122.00 ± 1.70	294.30 ± 2.00	33.00 ± 1.20
Negative control	284.00 ± 1.20^*^	208.00 ± 4.60^*^	392.30 ± 1.50^*^	42.00 ± 0.60^*^
AF2240-i	249.00 ± 13.90^*^	156.00 ± 2.30^**^	339.00 ± 1.70^**^	39.30 ± 0.30^*^
rAF-IL12	209.30 ± 0.58	148.00 ± 1.70^**^	316.00 ± 7.50^***^	39.00 ± 1.60^*^

Data are presented as mean ± S.E.M from six mice per group. Mean values with statistical difference at p ≤ 0.05 between groups are indicated with *

### Histopathological assessment of lung, spleen, kidney, and liver

Histopathological assessment on lung, spleen, kidney, and liver were carried out at the end of the study (day-28) to observe any abnormalities in the negative control (untreated), AF-2240-I, and rAF-IL12 groups in comparison to the respective organs in normal group (Additional files 4, 5, 6 and 7: Figures S4–S7). Histopathological assessment of the lungs of rAF-IL12 and untreated groups showed normal alveoli structure, comparable to the lungs in normal group but had mild thickening of alveolar interstitial wall due to leucocytic infiltration (Additional file 4: Figure S4). Leucocytic infiltrations were also observed in the lungs of AF2240-i-treated group, which caused pronounced thickening of alveolar interstitial wall. Histopathological assessment of spleen from CT26-tumour burden mice treated with AF2240-i and rAF-IL12 showed a normal architecture of spleen with distinct separation of white pulp and red pulp structure (Additional file 5: Figure S5). However, degeneration in white pulp area and depletion of lymphocyte as well as poor distinction between the red pulp and the white pulp were observed in the spleen of the untreated group. As shown in Additional file 6: Figure S6, the respective groups of CT26 tumour-burden mice treated with AF2240-i and rAF-IL12 retained their normal kidney architecture (i.e. comparable to the normal group), without obvious pathological lesions although there was leucocytic infiltration observed in AF2240-i. Meanwhile, the negative control/untreated group had an abnormal form of renal corpuscle with the size of Bowman’s space appeared to be smaller than kidney from the other groups. Leucocytic infiltration in the interstitial space of kidney was also observed in the untreated group. As shown in Additional file 7: Figure S7, the liver of the CT26 tumour-burden mice treated with rAF-IL12 did not show any obvious degeneration of hepatocytes or liver damage. However, there were some hepatocytes undergone apoptosis in the liver of the untreated and AF2240-i groups. Liver metastasis was observed in the untreated group as well as in the liver of mice treated with AF2240-i and rAF-IL12. There was also leucocytic infiltration present at the central vein of liver of the three aforementioned groups. These observations suggest that treatment with rAF-IL12 did not affect or cause abnormalities towards lung, spleen, kidney, and liver.

## Discussion

NDV has been extensively studied for its oncolytic effects in different tumour cell lines and in vivo models but have shown limited toxicity to normal cells [9, 25]. For instance, intra-tumoral treatment with naturally occurring strains of NDV, NDV/Altai/pigeon/770/2011 resulted in regression of human lung tumour growth in severe combined immunodeficient (SCID) mice while in another study, NDV-AF2240 strain showed its anti-tumoral effects by suppressing the growth of solid breast tumour in mice [26, 27]. At present, genetic engineering technology has been shown to boost the therapeutic activity of the virus against cancer, making the prospect of eradicating cancer seems more promising. In this study, the cytotoxicity effects and oncolytic properties of recombinant NDV, rAF-IL12 on CT26 murine colon cancer cell line were evaluated. The recombinant NDV, rAF-IL12 is the product of IL-12 insertion into the negative-sense RNA genome of wild-type NDV, AF2240-i which have shown to have similar replicative capability to its parental NDV strain as evaluated by the Taqman RT-qPCR analysis. The ability of these NDVs replicating in and targeting CT26 cells is due to the defects in anti-viral signalling pathways harboured by neoplastic cells making them highly susceptible to NDV [28]. This study postulated that the rAF-IL12 had better cytotoxicity effects (i.e. more potent) when compared to the AF2240-i NDV, as can be seen in the in vitro assays such as MTT assay (lower IC_50_ value), Annexin V FITC analysis (higher percentage of apoptotic cells as measured under the basis of the binding of FITC-conjugated Annexin V towards exposed phosphatidylserine (PS) on the cell membrane surface at the onset of apoptosis) [29], and cell cycle analysis (higher percentage of cell cycle arrest at G_1_ checkpoint).

The results from in vitro studies demonstrated the cytotoxic potential of rAF-IL12 against cancer cells. To further strengthen the justification of incorporating interleukin-12 in the negative-sense RNA genome of the wild-type NDV, AF2240-i, in vivo evaluation was carried out. Treatment of the colon cancer-challenged mice with rAF-IL12 resulted in a significant (p < 0.05) decrease in tumour weight (4.16-fold) and volume (6.32-fold) when compared to the untreated group while AF2240-i treatment only exhibited a much lower reduction fold in weight and volume of the tumour (2.14-fold and 3.05-fold, respectively). It was in agreement with the previous study which showed the recombinant NDV elicited better anti-tumoral effects than the wild-type [30]. Despite that, it is imperative for any cancer therapies including oncolytic virus to possess therapeutic efficacy without failing to look for potential harm towards patients. It has been elucidated that the use of interferon-sensitive viruses likes NDV would offer a broader safety margin for cancer treatment compared to chemotherapy [31]. NDV tumour specificity is based on cancer-specific defects in interferon pathway, which would only target and kill tumour cells and have mercy on normal cells [32, 33]. In this study, rAF-IL12 treatment was delivered through intra-tumoral injection as it is a routine method for local viral therapy in tumour tissues which might reduce systemic toxicity [34]. Although the virus was notably present in the lung, liver, kidney, and spleen, the viral copy number in those tissues were significantly lower when compared to the tumours (Additional file 3: Figure S3) and would not pose long-term toxicity problem considering the rAF-IL12 copy number was decreasing over time (Additional files 1 and 2: Figures S1–S2). This was a result of the virus elimination through functional interferon pathway in the normal tissues. Dissemination of rAF-IL12 virus in the tissues was deemed relatively safe as histopathological assessment of those organs revealed that rAF-IL12 treatment did not cause any observable pathological lesions or degeneration. This is because all the organs retained their normal morphology (i.e. lung: normal alveolar morphology but with mild thickening of alveolar interstitial wall due to leucocytic infiltration; spleen: normal spleen morphology with distinct separation of white pulp and red pulp structure; kidney: normal kidney architecture; liver: no obvious degeneration of hepatocytes or liver damage). The safety concerns regarding the potential usage of rAF-IL12 as oncolytic virotherapy was further evaluated by serum biochemistry profile analysis for liver and kidney functions. Previous studies have shown that increased level of aspartate aminotransferase (AST), alkaline phosphatase (ALP), and alanine aminotransferase (ALT) indicate acute hepatocellular injury while high creatinine level is an indicator for kidney injury [35, 36]. In this study, the untreated/negative control tumour-burden mice showed an elevated level of enzymes (AST, ALP, ALT, and creatinine) compared to the normal mice group. However, treatment with rAF-IL12 managed to reduce the enzymes level back to almost similar level to normal mice (i.e., better than AF2240-i) hence, suggesting that the virus did not result in tissues injury or compromising their function.

In addition to the direct oncolysis effect of the virus, the significant (p < 0.05) reduction in tumour growth was also due to the fact that NDV is an immunomodulatory agent, which mediates the activation of immune cells such as natural killer (NK) cells, macrophages, and cytotoxic T-lymphocytes [9, 12, 37]. The interleukin-12-encoding NDV demonstrated greater activation of CD4 + T-helper cells and CD8 + cytotoxic T-cells and drove a more profound secretion of IL-12, IL-2, and IFN-γ cytokines compared to the parental virus. CD8 + cytotoxic T-cells and NK cells serves as the first line of defense of immune system (i.e., innate immunity), acting upon cancer cells by releasing perforin and granzymes, which causes cell death [38, 39]. Previous study has demonstrated that the introduction of functional hemagglutinin-neuraminidase (HN) molecules by NDV on the surface of tumour cells facilitates the CD8 + T-cell response through their cell binding and the HN could also act as a ligand for NKp46 and NKp44 receptors on human NK cells, directly activating NK cells and contributing to the anti-tumoral effect of NDV [37, 40]. As the cells are dying, they would release the tumour associated antigens (TAA) and danger signals called damage-associated molecular patterns (DAMPs) and pathogen-associated molecular patterns (PAMPs), which triggers the activation of antigen-presenting cells (APCs) such as macrophages and dendritic cells [41]. The now activated APCs engulf any foreign particles including the tumour cells and presents the antigen at their major histocompatibility complex II (MHCII) for recognition and engagement with CD4 + T-helper cells [42]. The engagement results in the release of IL-12 by the APCs, which signals subsequent production and secretion of IL-2 and IFN-γ by the actively function CD4 + T-helper cells [43–45]. The release of IL-2 and IFN-γ amplifies the immune response as IL-2 functions in delivering signals for activation, maturation, and differentiation of T-cells, B-cells and NK cells while IFN-γ recruits and activates more NK cells to the tumour microenvironment [46, 47]. In this light, it was interesting to note the relevance of incorporating IL-12 into the negative-sense RNA genome of the NDV given that it is one of the core immune response inducers and would provide a higher expression level of IL-12 through the viral replication machinery inside tumour cells (i.e., exogenous IL-12 cytokines production was independent to that of the matured APCs secretion). This will lead to enhanced functionality in terms of generation/amplification of other cytokines (IL-2 and IFN-γ) and also directly augments end effectors such as CD8 + T-cells and NK cells (in comparison to natural anti-tumour immunity or induced by parental NDV) hence, further increases the immune response against cancer cells.

Tumourigenesis is associated with the over-proliferation of cells and the decrease of apoptosis and for that reason has become the main issue/subject to be tackled by any cancer treatment. CT26 cell line is a typical example of colon cancer expressing mutant KRAS that is associated with the degree of aggressiveness of colon cancer due to its importance in affecting growth, proliferation, and differentiation of cells through mitogen-activated protein kinase/extracellular signal-regulated kinase (MAPK/ERK) signaling cascade [48]. In addition to KRAS, other components like BRAF, MEK, and MAPK1 are also involved and mutation in one of them leads to a constitutive activation of MAPK/ERK signaling pathway that ultimately results in neoplasm [49–51]. Treatment of colon-cancer challenged mice with rAF-IL12 had resulted in the downregulation of KRAS, BRAF, and MAPK1 expression in tumour (Fig. 10b) while previous studies had reported that expression inhibition of these genes would improve patient survival [50, 51]. Intriguingly, rAF-IL12 did not downregulate the expression of KRAS and MAPK1 in CT26 cancer cell line as their expression level was similar to the untreated and AF2240-i (Fig. 10a). It is plausible considering that in vivo studies allowed rAF-IL12 to cause direct oncolysis as well as eliciting immune response towards tumours whereas in cell line, rAF-IL12 only targeted cancer cells by its direct oncolysis. Additionally, histopathology of tumour tissues showed abundance of mitotic features in the untreated and AF2240-i-treated groups indicating rampant tumour growth whereas rAF-IL12 treatment had significantly (p < 0.05) reduced the number of mitotic cells. Furthermore, rAF-IL12 also exhibited its apoptotic activity as it significantly (p < 0.05) reduced the expression of NOTCH-1 in vivo; NOTCH-1 signaling pathway is involved in pathogenesis of colorectal cancer [52]. Previous study showed that inhibition of NOTCH-1 by lentiviral-encoding NOTCH-1 siRNA can effectively suppress the growth and proliferation of colon cancer cells and promote cell apoptosis [53]. rAF-IL12 also mediated p53-apoptosis pathway as it increased the expression level of BAX and p53 in vitro and in vivo. BAX, which belongs to bcl-2 family, is a tumour suppressor and an essential effector of the mitochondrial apoptotic pathways whereby its activation by p53 (best known for its role in causing cell cycle arrest or apoptosis) signal results in the release of cytochrome c and second mitochondria-derived activator of caspases/direct inhibitor of apoptosis-binding protein with low pI (Smac/DIABLO) from mitochondria, which facilitates caspase activation and subsequent nuclear fragmentation [54, 55]. Moreover, it has been shown that the AF2240 NDV infection promotes BAX translocation from cytoplasm to mitochondria and caused cell death in HeLa cells thus suggesting the same mechanism adopted by the rAF-IL12 [56]. Other than that, the apoptotic activity of rAF-IL12 was also shown through TUNEL assay as the number of cancer cells undergone apoptosis (TUNEL-positive cells) were significantly (p < 0.05) higher than the other two groups. TUNEL assay was used to detect nuclear DNA fragmentation by identifying generation of nicks and breaks in the DNA strands caused by rAF-IL12 treatment [57].

Anti-metastatic potential of rAF-IL12 had been also elucidated in this study through the histopathological assessment of liver in all colon cancer-challenged mice groups whereby liver metastases were present in all groups. However, the population size of neoplastic tumour cells in the rAF-IL12 treated group was the smallest compared to the ones in untreated and AF2240-i treated groups. It might be due to the decreased expression level of CCL2 chemokine; small peptides that are structurally and functionally similar to growth factors that could induce chemoattraction, inflammation, and promote cancer cell metastasis, as well as angiogenesis [58, 59]. Other than that, rAF-IL12 also exhibited anti-angiogenic properties as the expression level of vascular endothelial growth factor-A (VEGF-A) was significantly (p < 0.05) decreased compared to the negative control and AF2240-i treated groups. VEGF-A is the predominant angiogenic factor in CRC as it is expressed in approximately 50% of CRC. The downregulation of VEGF consequently impedes the generation of new capillaries from pre-existing blood vessels at the surrounding matrix. Hence, inhibiting the nutrients supply for tumour growth and development as well as halting their progression (i.e. VEGF-expressing tumours had greater vascularity and metastatic potential [60, 61].

However, there are few limitations regarding the use of this recombinant NDV, rAF-IL12 as anti-cancer vaccine in targeting colon cancer. Viral dissemination into other organs from the point of intra-tumoral injection is a major concern that needs to be tackled as it may reduce the efficacy of the viral delivery and oncolytic effects of rAF-IL12 towards tumour. As this study demonstrated the potential of rAF-IL12 in inhibiting the progressive growth of CT26 tumour through the intra-tumoral injection, it has raised question over its efficacy potential when administered via other routes such as intra-venous. Of note, intra-venous injection is considered the most appropriate way to deliver the viral anti-cancer vaccine in patients but it also presents a challenge as the systemic administration requires large therapeutic doses to ensure for adequate delivery to the tumour site and carries safety implications such as systemic inflammatory response and off-target toxicity [62]. Furthermore, the rAF-IL12 should be tested on other types of colon cancer cell lines to provide information on its exclusive cytotoxic potential against colon cancer.

## Conclusion

Based on this study, rAF-IL12 managed to impede the growth of CT26 tumour in a murine model in vivo. Additionally, rAF-IL12 was also able to induce apoptosis in vitro and in vivo, increased the expression level of apoptosis-related genes, and decreased the expression level of oncogenes as well as regulated the immune response in targeting the CT26 tumours. Moreover, rAF-IL12 intra-tumoral delivery was considered safe and not hazardous to the host as evidenced in the pathophysiology of the normal tissues and organs of the mice as well as from the serum biochemistry profile of liver and kidney. As the rAF-IL12 shows potential anti-cancer effect on the CT26 colon cancer model, additional studies including testing on other colon cancer cells and detailed protein expression profiling in the future studies will strengthen the preclinical understanding of the efficacy and mechanism of rAF-IL12, to better prepare the translational application to the future clinical usage as anti-colon cancer vaccine.

## Supplementary information

**Additional file 1: Figure S1.** Viral replication kinetics of **a** AF2240-i and **b** rAF-IL`12 based on the viral copy number in lung, spleen, liver, and kidney of normal Balb/c mice as determined by real-time PCR analysis after each time-point (day-2, day-3, day-7, and day-10). Data are presented as mean ± S.E.M from six mice per group. Statistically significant differences between the means were determined by One-Way ANOVA followed by Duncan post hoc test. Differences were considered significant when the *p ≤ 0.05.

**Additional file 2: Figure S2.** Viral replication kinetics of **a** AF2240-i and **b** rAF-IL12 based on the viral copy number in the tumour, lung, spleen, liver, and kidney of the CT26 colon cancer-challenged Balb/c mice as determined by real-time PCR analysis at day-2, day-3, and day-7 time-point. Data are presented as mean ± S.E.M from six mice per group. Statistically significant differences between the means were determined by One-Way ANOVA followed by Duncan post hoc test. Differences were considered significant when the *p ≤ 0.05.

**Additional file 3: Figure S3.** Viral copy number inside the tumour, lung, spleen, liver, and kidney (at day-28) of the AF2240-i-treated and rAF-IL12-treated groups of the CT26 colon cancer-challenged mice study as determined by real-time PCR analysis. Data are presented as mean ± S.E.M from six mice per group. Statistically significant differences between the means were determined by One-Way ANOVA followed by Duncan post hoc test. Differences were considered significant when the *p ≤ 0.05.

**Additional file 4: Figure S4.** Photomicrograph section of the lung of mice stained with H&E from 4 different groups of mice **a** Normal, **b** Untreated, **c** AF2240-i-treated, and **d** rAF-IL12-treated. Normal group showed normal alveolar morphology; alveolar air space (green arrow) and alveolar capillary (yellow arrow). Untreated and rAF-IL12-treated showed normal alveolar morphology; alveolar air space (green arrow) and alveolar capillary (yellow arrow) but with mild thickening of the alveolar interstitial wall due to leucocytic infiltration (blue arrow). AF2240-i-treated showed pronounced thickening of the alveolar interstitial wall due to leucocytic infiltration (blue arrow). *AV* alveolar duct, *V* vein, *BR* bronchiole, *AV* alveoli. Magnification: 100X; H&E scale bar = 200 µm.

**Additional file 5: Figure S5.** Photomicrograph of the spleen of mice stained in H&E from 4 different groups; (A) Normal, (B) Untreated, (C) AF2240-i-treated, and (D) rAF-IL12-treated. Spleen from (A, C, and D) groups showed no pathological changes with distinct white pulp and red pulp structure. Note the lymphocyte depletion (yellow arrow) in the white pulp and poor distinction of the white pulp from the red pulp in (B) group. WP, white pulp; RP, red pulp; CA, central artery; GC, germinal centre; PALS, periarteriolar lymphoid sheaths. Magnification: 100 × ; H&E scale bar = 200 µm.

**Additional file 6: Figure S6.** Photomicrograph section of kidney stained with H&E from 4 different groups of mice, **a** Normal, **b** Untreated, **c** AF2240-i-treated, and **d** rAF-IL12-treated. Note the leucocytic infiltration in the interstitial space (black arrow) in (**b** and **c**) and the size of Bowman’s space became smaller in (**b**). *RC* renal corpuscle with glomeruli, *BS* Bowman’s space, *BC* Bowman’s capsule, *p* proximal tubule, *d* distal tubule. Magnification: 400X; H&E scale bar = 50 µm.

**Additional file 7: Figure S7.** Photomicrograph of mouse liver stained with H&E from 4 groups of mice; **a** Normal, **b** Untreated, **c** AF2240-i-treated, and **d** rAF-IL12-treated. Normal hepatocytes with obvious central vein shown in (**a**). Note the anaplastic tumour cells with cellular and nuclear variation in shape and size (blue arrow) in (**b**), liver metastasis (yellow arrow) in (**b, c** and **d**), the hepatocellular apoptosis (blue block arrow) in (**b** and **c**), and inflammatory infiltrates (green arrow) in (**b**, **c** and **d**). *S* blood sinusoids, *CV* central vein. Magnification: 400X; H&E scale bar = 50 µm.

## Data Availability

All data generated or analysed during this study are included in this published article.

## Abbreviations

ALP: Alkaline phosphates
ALT: Alanine aminotransferase
APC: Antigen-presenting cells
AST: Aspartate aminotransferase
Avidin-HRP: Avidin-horse radish peroxidase
BAX: BCL2 associated X
BSA: Bovine serum albumin
CCL2: C-C motif chemokine ligand 2
DAMP: Damage-associated molecular patterns
DMSO: Dimethyl sulfoxide
HA: Haemagglutinin assay
HN: Hemagglutinin-neuraminidase
H&E: Hematoxylin and Eosin
IACUC: International Animal Care and Use Committee
IFN-γ: Interferon gamma
IL-2: Interleukine-2
IL-12: Interleukine-12
KRAS: Kirsten rat sarcoma
MAPK1: Mitogen-activated protein kinase 1
MAPK/ERK: Mitogen-activated protein kinase/extracellular signal-regulated kinase
MHC II: Major histocompatibility complex II
MTT: 3- [4,5-dimethylthiazol-2-yl]-2,5 diphenyltetrazolium bromide
NDV: Newcastle disease virus
NK: Natural killer
PAMP: Pathogen-associated molecular patterns
PBS: Phosphate-buffered saline
SCID: Severe combined immunodeficient
Smac/DIABLO: Second mitochondria-derived activator of caspases/direct inhibitor of apoptosis-binding protein with low pI
TAA: Tumour associated antigens
TMB: 3,3′,5,5′- tetramethylbenzidine
TUNEL: Terminal deoxynucleotide transferase (TdT) dUTP Nick-End Labeling
VEGF-A: Vascular endothelial growth factor-A
