# Supplementary figures and images for: Cytotoxicity study of the interleukin-12-expressing recombinant Newcastle disease virus strain, rAF-IL12, towards CT26 colon cancer cells in vitro and in vivo
Source: Cancer Cell Int. 2020 Jun 29;20:278. doi: 10.1186/s12935-020-01372-y (PMC7325054; doi:10.1186/s12935-020-01372-y)

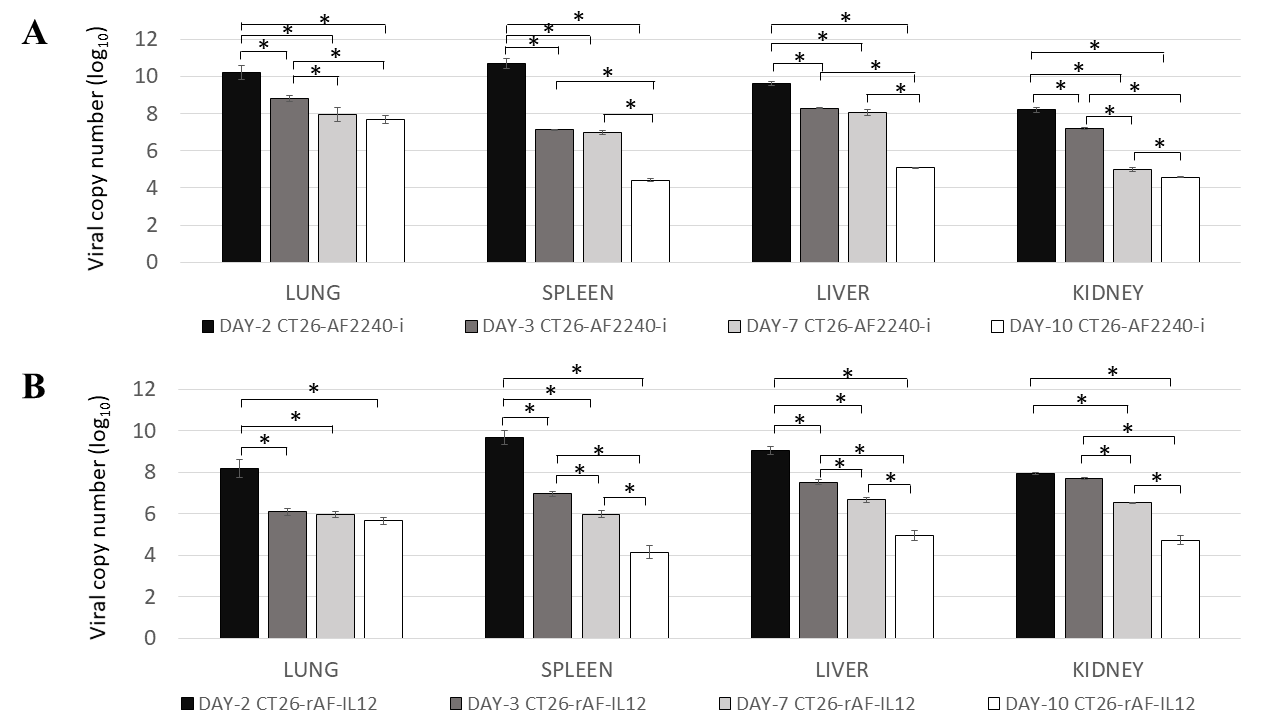

Supplement: Supplementary file 1 — Additional file 1: Figure S1. Viral replication kinetics of a AF2240-i and b rAF-IL`12 based on the viral copy number in lung, spleen, liver, and kidney of normal Balb/c mice as determined by real-time PCR analysis after each time-point (day-2, day-3, day-7, and day-10). Data are presented as mean ± S.E.M from six mice per group. Statistically significant differences between the means were determined by One-Way ANOVA followed by Duncan post hoc test. Differences were considered significant when the *p ≤ 0.05. [file 12935_2020_1372_MOESM1_ESM.tif]

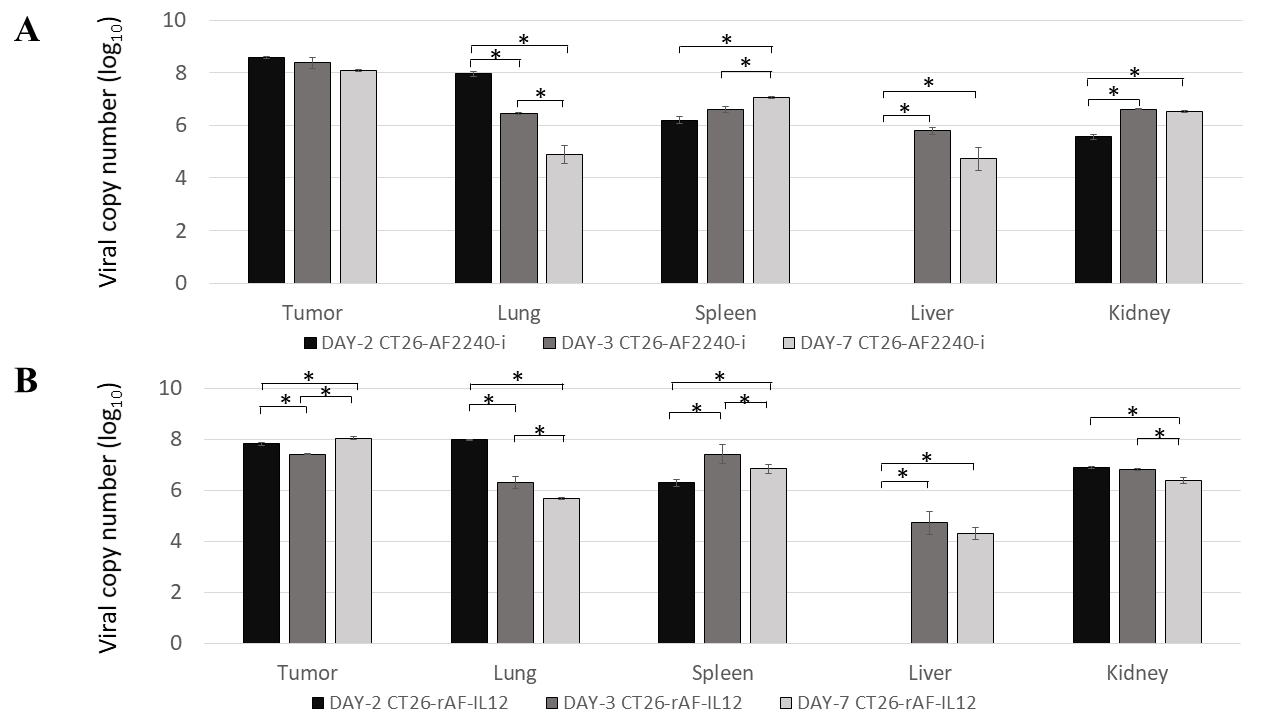

Supplement: Supplementary file 2 — Additional file 2: Figure S2. Viral replication kinetics of a AF2240-i and b rAF-IL12 based on the viral copy number in the tumour, lung, spleen, liver, and kidney of the CT26 colon cancer-challenged Balb/c mice as determined by real-time PCR analysis at day-2, day-3, and day-7 time-point. Data are presented as mean ± S.E.M from six mice per group. Statistically significant differences between the means were determined by One-Way ANOVA followed by Duncan post hoc test. Differences were considered significant when the *p ≤ 0.05. [file 12935_2020_1372_MOESM2_ESM.tif]

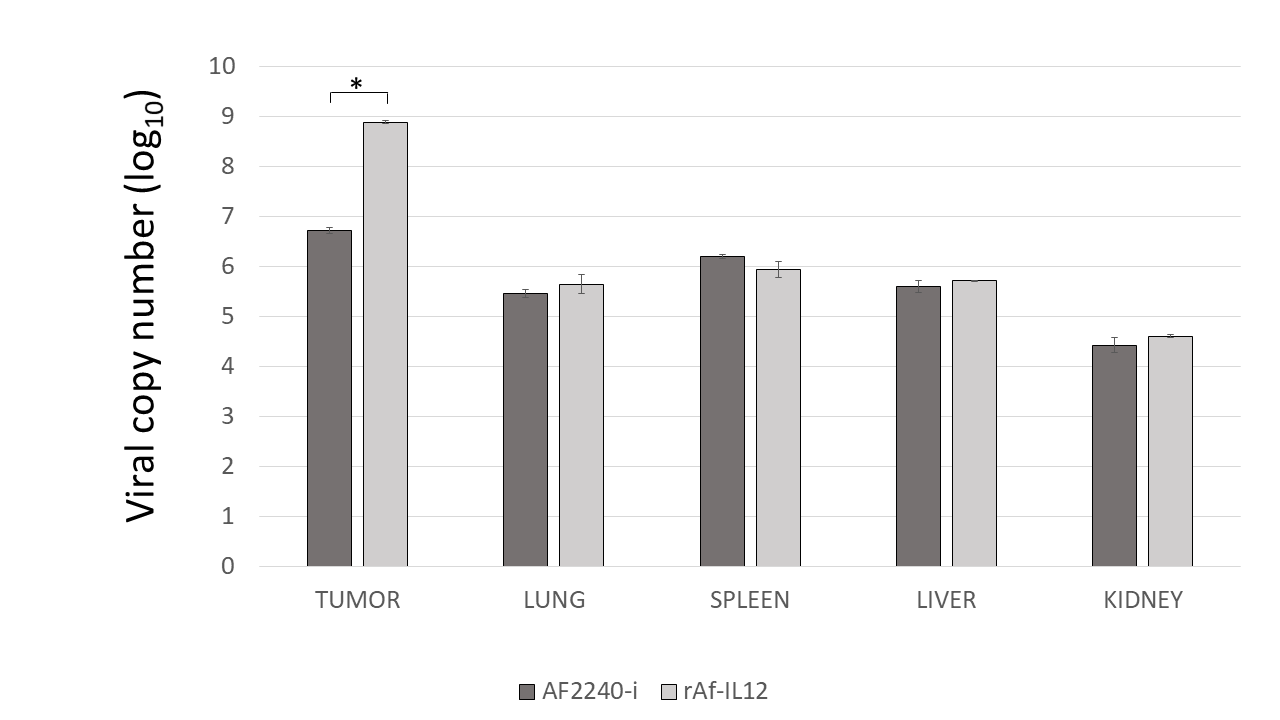

Supplement: Supplementary file 3 — Additional file 3: Figure S3. Viral copy number inside the tumour, lung, spleen, liver, and kidney (at day-28) of the AF2240-i-treated and rAF-IL12-treated groups of the CT26 colon cancer-challenged mice study as determined by real-time PCR analysis. Data are presented as mean ± S.E.M from six mice per group. Statistically significant differences between the means were determined by One-Way ANOVA followed by Duncan post hoc test. Differences were considered significant when the *p ≤ 0.05. [file 12935_2020_1372_MOESM3_ESM.tif]

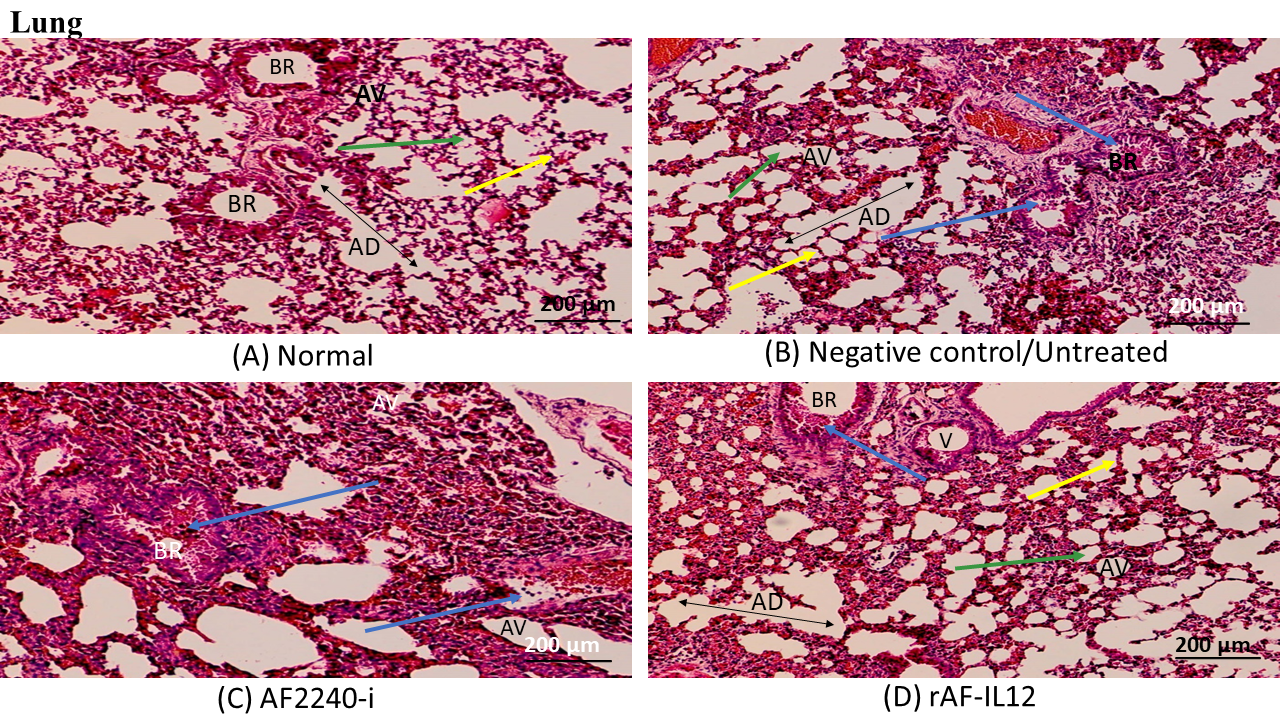

Supplement: Supplementary file 4 — Additional file 4: Figure S4. Photomicrograph section of the lung of mice stained with H&E from 4 different groups of mice a Normal, b Untreated, c AF2240-i-treated, and d rAF-IL12-treated. Normal group showed normal alveolar morphology; alveolar air space (green arrow) and alveolar capillary (yellow arrow). Untreated and rAF-IL12-treated showed normal alveolar morphology; alveolar air space (green arrow) and alveolar capillary (yellow arrow) but with mild thickening of the alveolar interstitial wall due to leucocytic infiltration (blue arrow). AF2240-i-treated showed pronounced thickening of the alveolar interstitial wall due to leucocytic infiltration (blue arrow). AV alveolar duct, V vein, BR bronchiole, AV alveoli. Magnification: 100X; H&E scale bar = 200 µm. [file 12935_2020_1372_MOESM4_ESM.tif]

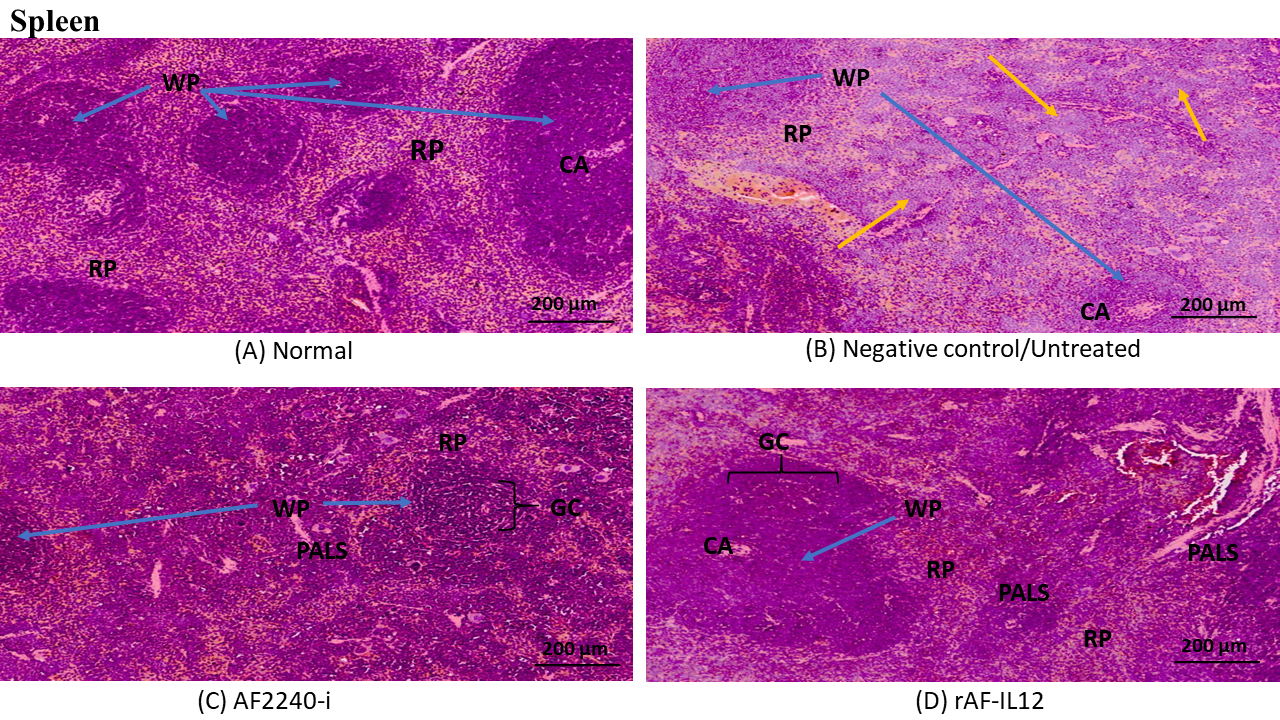

Supplement: Supplementary file 5 — Additional file 5: Figure S5. Photomicrograph of the spleen of mice stained in H&E from 4 different groups; (A) Normal, (B) Untreated, (C) AF2240-i-treated, and (D) rAF-IL12-treated. Spleen from (A, C, and D) groups showed no pathological changes with distinct white pulp and red pulp structure. Note the lymphocyte depletion (yellow arrow) in the white pulp and poor distinction of the white pulp from the red pulp in (B) group. WP, white pulp; RP, red pulp; CA, central artery; GC, germinal centre; PALS, periarteriolar lymphoid sheaths. Magnification: 100 × ; H&E scale bar = 200 µm. [file 12935_2020_1372_MOESM5_ESM.tif]

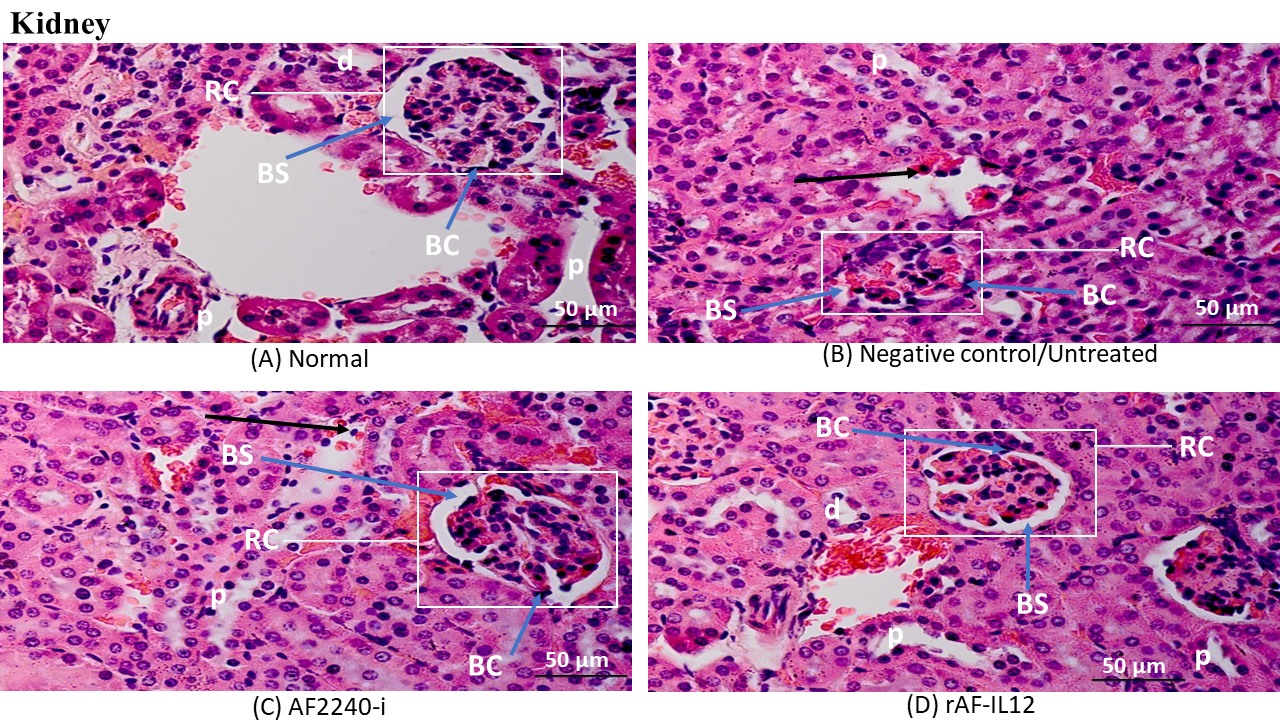

Supplement: Supplementary file 6 — Additional file 6: Figure S6. Photomicrograph section of kidney stained with H&E from 4 different groups of mice, a Normal, b Untreated, c AF2240-i-treated, and d rAF-IL12-treated. Note the leucocytic infiltration in the interstitial space (black arrow) in (b and c) and the size of Bowman’s space became smaller in (b). RC renal corpuscle with glomeruli, BS Bowman’s space, BC Bowman’s capsule, p proximal tubule, d distal tubule. Magnification: 400X; H&E scale bar = 50 µm. [file 12935_2020_1372_MOESM6_ESM.tif]

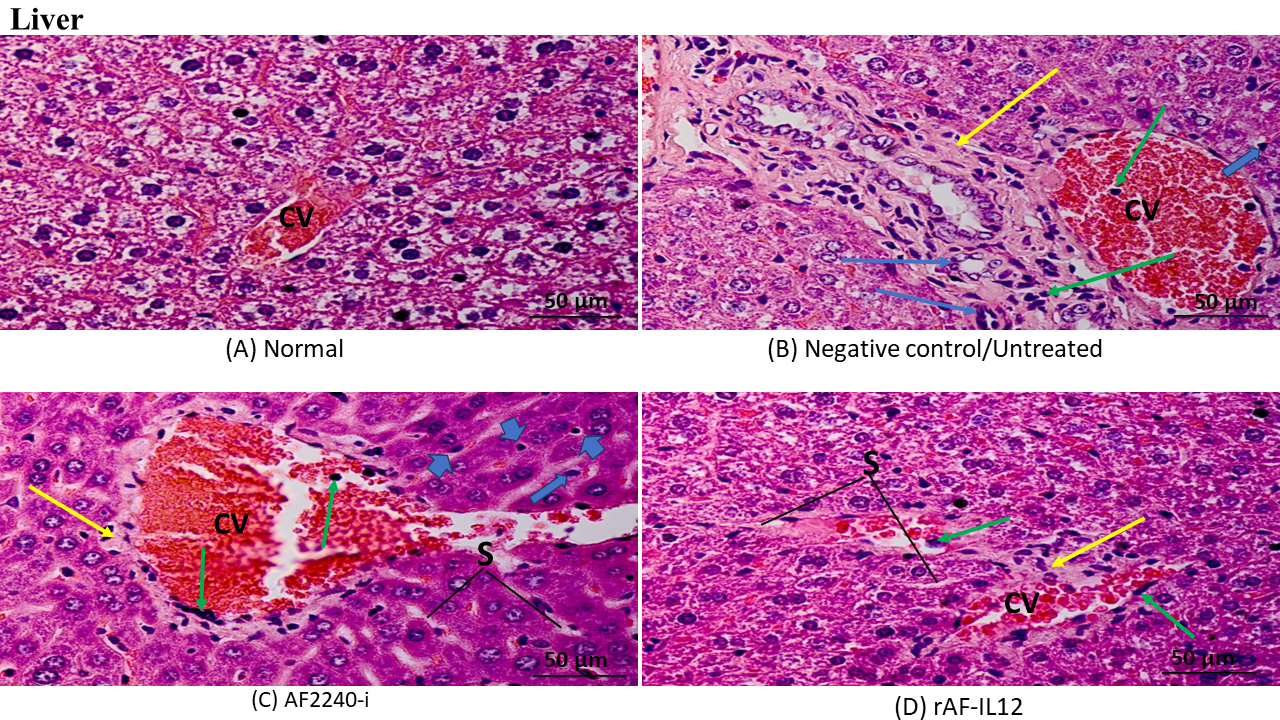

Supplement: Supplementary file 7 — Additional file 7: Figure S7. Photomicrograph of mouse liver stained with H&E from 4 groups of mice; a Normal, b Untreated, c AF2240-i-treated, and d rAF-IL12-treated. Normal hepatocytes with obvious central vein shown in (a). Note the anaplastic tumour cells with cellular and nuclear variation in shape and size (blue arrow) in (b), liver metastasis (yellow arrow) in (b, c and d), the hepatocellular apoptosis (blue block arrow) in (b and c), and inflammatory infiltrates (green arrow) in (b, c and d). S blood sinusoids, CV central vein. Magnification: 400X; H&E scale bar = 50 µm. [file 12935_2020_1372_MOESM7_ESM.tif]
